# Identification of carbon-encapsulated iron nanoparticles as active species in non-precious metal oxygen reduction catalysts

**DOI:** 10.1038/ncomms12582

**Published:** 2016-08-19

**Authors:** Jason A. Varnell, Edmund C. M. Tse, Charles E. Schulz, Tim T. Fister, Richard T. Haasch, Janis Timoshenko, Anatoly I. Frenkel, Andrew A. Gewirth

**Affiliations:** 1 Department of Chemistry, University of Illinois at Urbana–Champaign, Urbana, Illinois 61801, USA; 2 Department of Physics, Knox College, Galesburg, Illinois 61401, USA; 3 Chemical Sciences and Engineering Division, Argonne National Laboratory, Argonne, Illinois 60439, USA; 4 Frederick Seitz Materials Research Laboratory, University of Illinois, Urbana, Illinois 61801, USA; 5 Department of Physics, Yeshiva University, New York, New York 10016, USA; 6 International Institute for Carbon Neutral Energy Research (WPI-I2CNER), Kyushu University, Fukuoka 812-8581, Japan

## Abstract

The widespread use of fuel cells is currently limited by the lack of efficient and cost-effective catalysts for the oxygen reduction reaction. Iron-based non-precious metal catalysts exhibit promising activity and stability, as an alternative to state-of-the-art platinum catalysts. However, the identity of the active species in non-precious metal catalysts remains elusive, impeding the development of new catalysts. Here we demonstrate the reversible deactivation and reactivation of an iron-based non-precious metal oxygen reduction catalyst achieved using high-temperature gas-phase chlorine and hydrogen treatments. In addition, we observe a decrease in catalyst heterogeneity following treatment with chlorine and hydrogen, using Mössbauer and X-ray absorption spectroscopy. Our study reveals that protected sites adjacent to iron nanoparticles are responsible for the observed activity and stability of the catalyst. These findings may allow for the design and synthesis of enhanced non-precious metal oxygen reduction catalysts with a higher density of active sites.

## Abstract (web summary)

Determining active species in non-precious metal catalysts for the oxygen reduction reaction remains a challenge due to catalyst heterogeneity. Here the authors perform gas-phase treatments on an iron-based catalyst to allow the identification of carbon-encapsulated iron nanoparticles as the active species.

## Graphical abstract

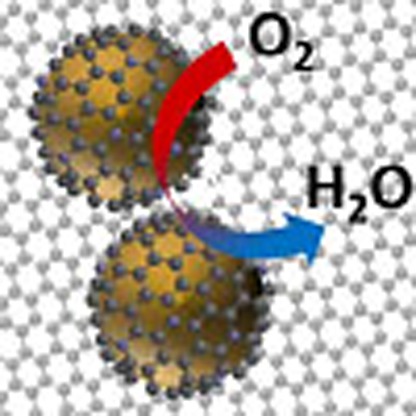

Fuel cells (FCs) offer a highly efficient method to convert chemical energy into electrical energy. Despite their advantages, fuel cells currently require the use of Pt-alloy catalysts for the oxygen reduction reaction (ORR), which makes such devices cost-prohibitive for many applications^1^,^2^. For this reason, non-precious metal (NPM) ORR catalysts have been intensely investigated, following the discovery of the ORR activity of Co phthalocyanines as early as 1964 (ref. 3). Pyrolysis of Fe- or Co-containing porphyrins and phthalocyanines affords catalysts of enhanced stability and activity^4^,^5^,^6^. Many other N-containing materials, when pyrolyzed in the presence of Fe salts, also exhibit promising activity^7^,^8^,^9^,^10^,^11^,^12^. Specifically, N-rich polymers such as polyaniline, lead to catalysts with ORR activity approaching that of Pt in acid^13^,^14^,^15^,^16^. However, progress towards making enhanced ORR materials is inhibited due to the lack of understanding regarding the ORR-active site.

Elucidating the nature of the catalytic centre in NPM ORR catalysts is challenging due to the heterogeneity introduced during the high-temperature synthesis that is also retained during ORR operation. Studies utilizing metal-binding ligands such as CN^−^ suggest that the activity in these catalysts is metal-centred^17^,^18^,^19^. Two models for the active site are suggested. The first model consists of a metal particle encapsulated by a carbon shell^20^,^21^. However, this model does not accommodate the requirement for nitrogen^5^,^7^,^22^. A second model invokes a porphyrin-like FeN_4_ (or FeN_2+2_) structure^23^,^24^,^25^,^26^. Studies of ORR-active materials show the presence of both metallic Fe and Fe–N species^16^,^24^,^27^,^28^. ORR catalysts containing a preponderance of either type of site have been recently synthesized, with catalysts suggested to feature FeN_4_ species exhibiting the highest activity to date^26^,^29^,^30^,^31^. However, the active species in the vast majority of reported NPM catalysts remains unknown due to the heterogeneity present.

Efforts to permanently deactivate a NPM ORR catalyst to locate the source of ORR activity have been unsuccessful. Treating the catalyst with strong acid does not remove all of the Fe metal and in fact leads to a more active catalyst^8^,^23^,^27^,^32^,^33^,^34^. The residual metal is assumed to be inactive for ORR, but the results are not definitive. Selective poisoning that could identify specific centres responsible for ORR activity is also problematic. NPM ORR catalysts exhibit a high tolerance to most anions, such as F^−^, SCN^−^, N_3_^−^ and phosphate^13^,^17^,^18^. Both pyrolyzed and unpyrolyzed NPM ORR catalysts are insensitive to CO and while the inhibitory effect of CN^−^ has been demonstrated, it can be removed by simply placing the CN^−^-exposed catalyst in fresh electrolyte without CN^−^ (refs 18, 35, 36). In contrast, the poisoning of the biological Fe heme-based ORR catalyst cytochrome *c* oxidase with CO has been observed^36^,^37^. The inconsistency in these results leaves room for further clarification into the active species found in the many pyrolyzed and unpyrolyzed ORR catalysts described in literature, including the role of the FeN_4_ site in pyrolyzed NPM catalysts due to the lack of poisoning that has been observed in some cases^37^,^38^. Interestingly, high-temperature treatments with H_2_S, H_2_ and NH_3_ have all increased NPM catalyst activity^10^,^25^,^26^,^32^,^39^. It has been noted that at high temperatures, NH_3_ would likely be decomposed to N_2_ and H_2_ (ref. 25). These conditions are reducing, but the chemical basis for this enhancement remains unclear^32^. We hypothesized that a high-temperature treatment in an oxidizing atmosphere such as Cl_2_ might be able to deactivate an NPM catalyst. Such treatments are used to oxidize and remove metallic impurities from carbon materials via the formation and sublimation of volatile metal chlorides^40^,^41^,^42^,^43^.

In this paper, we report the effects of high-temperature Cl_2_ and H_2_ treatments on a NPM ORR catalyst and show that a reproducible deactivation and reactivation of the catalyst are achieved via the respective treatments. In addition, we show that the heterogeneity of the catalyst is decreased by treatment with Cl_2_ and H_2_, which allows for the direct characterization of the species present in the deactivated and reactivated catalysts. Mössbauer spectroscopy, X-ray absorption near edge structure (XANES), extended X-ray absorption fine structure (EXAFS) and X-ray photoelectron spectroscopy (XPS) reveal the changes induced by the Cl_2_ and H_2_ treatments and clarify the nature of the ORR-active species.

## Results

### Effect of Cl_2_ and H_2_ treatments on ORR activity

For our work, we used a polyaniline-derived catalyst due to its reported high activity and stability, which we denote as the ‘as-prepared' catalyst^14^. We subjected the as-prepared catalyst material to a high-temperature treatment with Cl_2_ at 900 °C. The ‘Cl_2_-treated' catalyst was subsequently treated with H_2_ at 900 °C leading to the ‘H_2_-treated' catalyst.

**Figure 1:**
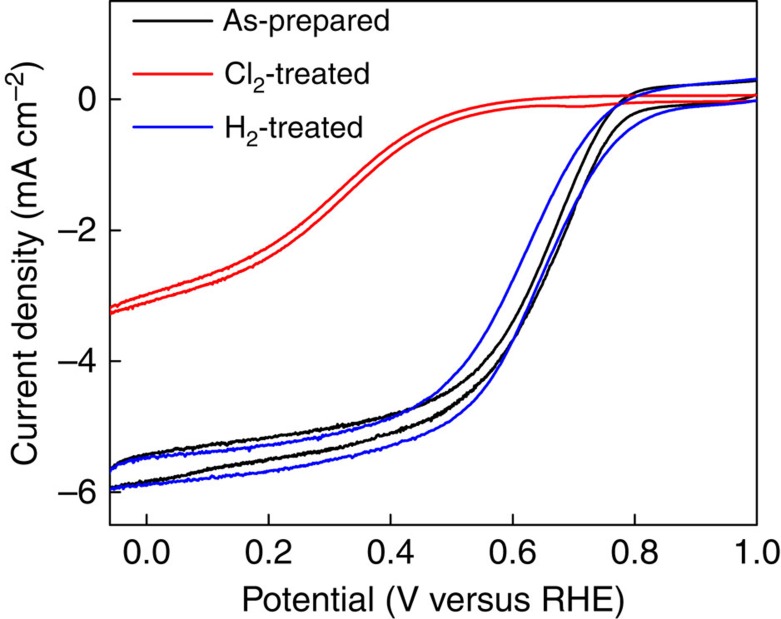
Electrochemical characterization of catalysts. Cyclic voltammograms of ORR on as-prepared, Cl_2_-treated and H_2_-treated catalysts in 0.1 M HClO_4_.

Figure 1 shows cyclic voltammetry (CV) on a rotating disk electrode (RDE) to evaluate the ORR activity of the three catalysts studied. The Cl_2_ treatment resulted in a negative shift of the ORR onset by ca. 170 mV. In addition, an increase in peroxide formation from 1.6 to 5.0% was observed after the Cl_2_ treatment using a rotating ring-disk electrode (RRDE) (Supplementary Fig. 1; Supplementary Table 1). These results indicate that the active site is poisoned or destroyed by the Cl_2_ treatment. After the H_2_ treatment, the ORR activity of the as-prepared catalyst was completely recovered, showing a similar onset potential and low peroxide yield. Repeated treatments with Cl_2_ and H_2_ on a single batch of as-prepared catalyst revealed that the observed effects are highly controllable (Supplementary Fig. 2).

**Figure 2:**
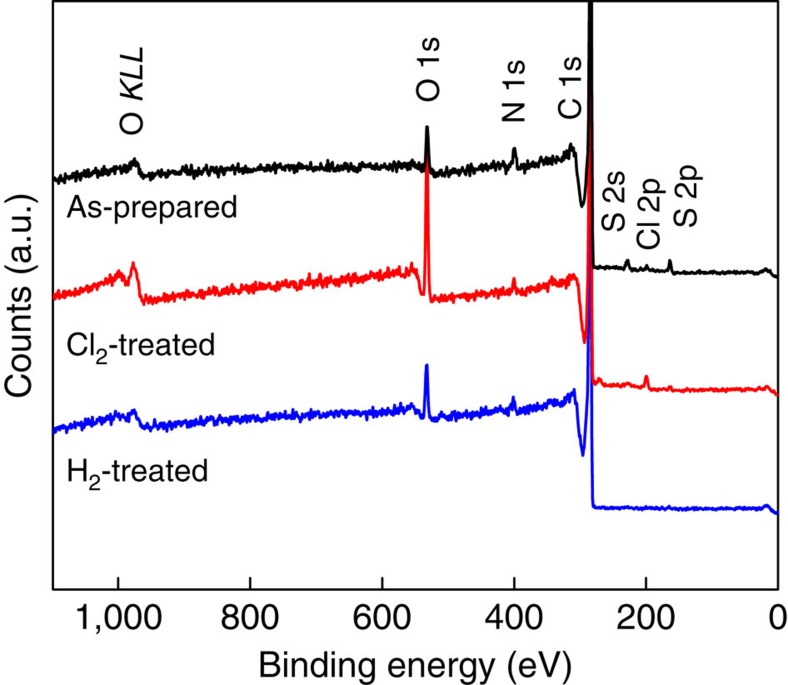
X-ray photoelectron spectroscopy. Spectra of as-prepared, Cl_2_-treated and H_2_-treated catalysts showing the surface species present on each catalyst studied.

To explain the observed deactivation and reactivation of the NPM material, we used inductively coupled plasma optical emission spectroscopy (ICP-OES) to measure the Fe content in the as-prepared, Cl_2_-treated and H_2_-treated materials (Supplementary Table 2). Although the Fe content was decreased by the Cl_2_ treatment, some Fe remained and was never completely removed, similar to previous attempts to demetalate the catalyst^8^,^28^. This result suggests that the form of the Fe present affects the observed ORR activity and not the total Fe content. The oxidizing nature of the Cl_2_ treatment favours the formation of oxidized Fe species, while the reducing nature of the H_2_ treatment causes the formation of reduced Fe species. In addition, the presence of Cl in the Cl_2_-treated catalyst was observed using elemental analysis (Supplementary Table 2). Survey XPS spectra are shown in Fig. 2 that also indicate the presence of Cl in the Cl_2_-treated sample. Supplementary Fig. 3 shows the high-resolution scans taken in the C 1 s region, which show no change in the surface carbon species present before and after the Cl_2_ and H_2_ treatments.

To investigate the effects of the treatment temperature, we treated the as-prepared catalyst with Cl_2_ at 600 °C and observed that no deactivation occurred (Supplementary Fig. 4), consistent with a prior report that found slight ORR activity enhancement following treatment with Cl_2_ at 650 °C (ref. 8). Supplementary Fig. 5 shows the XPS survey spectrum of the catalyst following 600 °C Cl_2_ treatment that indicates the presence of Cl similar to the 900 °C Cl_2_-treated catalyst. This suggests that the presence of Cl is not responsible for the large deactivation that was observed following Cl_2_ treatment at 900 °C on the as-prepared catalyst.

To determine if the deactivation observed following the Cl_2_ treatment at 900 °C was related to the presence of Fe, we synthesized the catalyst without Fe and carried out the same gas treatments at 900 °C. Supplementary Fig. 6 shows the electrochemical activity of the metal-free catalyst before and after the treatment with Cl_2_ and H_2_. The overall activity of the metal-free catalyst is significantly lower than that of the catalyst containing Fe, and only a small deactivation and reactivation of the metal-free catalyst was observed. Supplementary Figs 7 and 8 show the XPS survey spectra and C 1 s region for the metal-free catalyst that displays the same presence of Cl after Cl_2_ treatment and the removal of Cl with the H_2_ treatment that were observed for the Fe-based catalyst. Because of the small effect observed after Cl_2_ treatment and the low initial activity of the metal-free catalyst, the presence of the metal in the Fe-containing catalyst must contribute to the higher activity and large deactivation that were observed. To discover the nature of the Fe species in the as-prepared and treated catalysts, we utilized several additional methods to further examine each catalyst and determine the species responsible for the appreciable ORR activity observed.

### Mössbauer spectroscopy

**Figure 3:**
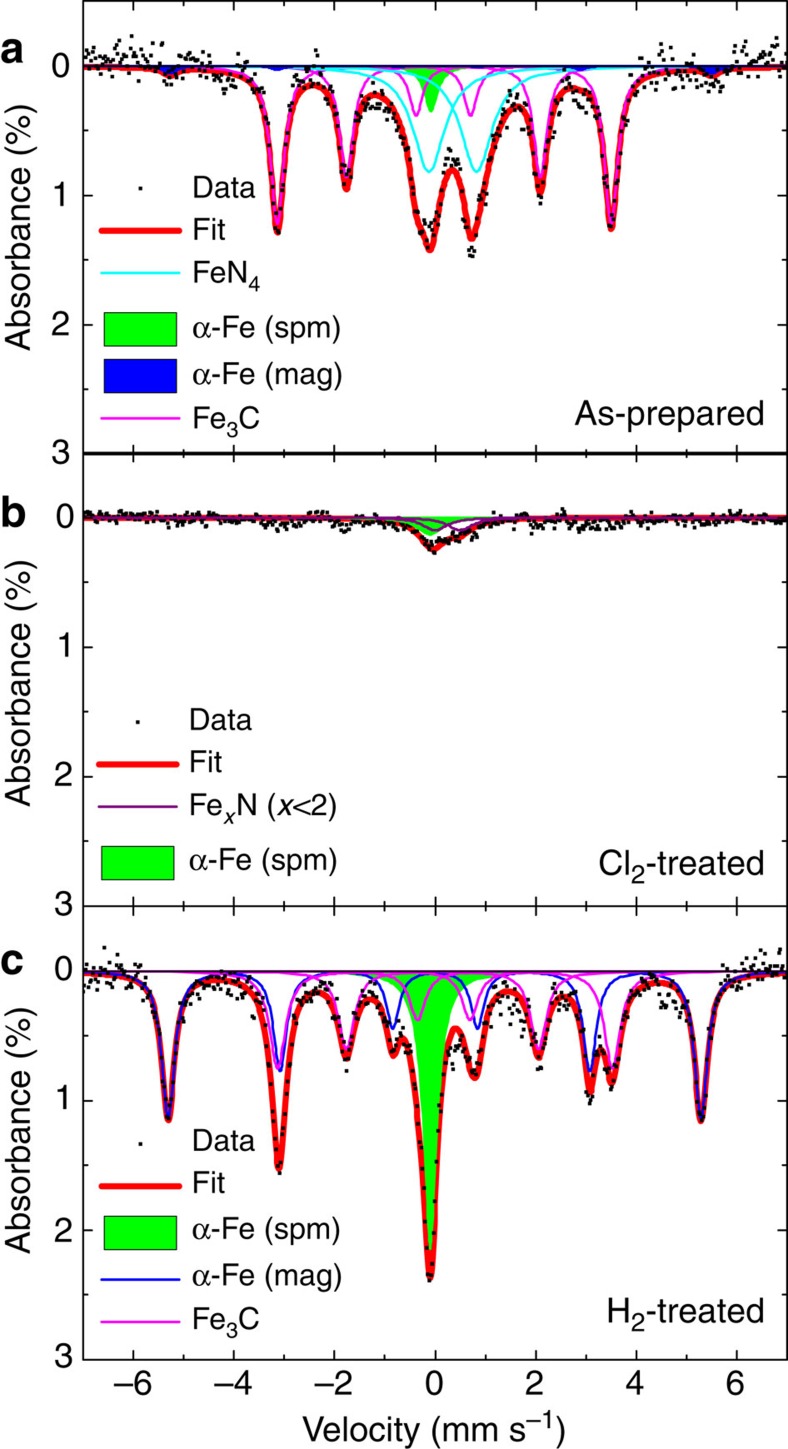
Mössbauer spectroscopy characterization of catalysts. Spectra and peak fitting of as-prepared (**a**), Cl_2_-treated (**b**) and H_2_-treated (**c**) catalysts at 300 K. Both magnetically split (mag) and superparamagnetic (spm) metallic Fe species are observed.

We evaluated the as-prepared and treated catalysts using Mössbauer spectroscopy (Fig. 3). Assignments for each of the signals observed for the three spectra are given in Supplementary Table 3 based on the previous literature. The Mössbauer spectrum of the as-prepared catalyst (Fig. 3a) shows FeN_4_ species along with Fe_3_C and α-Fe in the form of both magnetically split and superparamagnetic species, consistent with the previous literature^16^. The presence of both oxidized and reduced Fe species in the as-prepared catalysts inhibits identification of the active Fe species. The spectrum of the Cl_2_-treated catalyst (Fig. 3b) shows a small amount of Fe_*x*_N and superparamagnetic α-Fe, with a significantly smaller absorption area than for the as-prepared and H_2_-treated catalyst. The spectrum of the H_2_-treated catalyst (Fig. 3c) shows only reduced Fe species, including the magnetically split signal of α-Fe and a singlet from superparamagnetic α-Fe with additional contributions from Fe_3_C. Noticeably, absent in the H_2_-treated catalyst is the signature of any Fe–N species, including FeN_4_ and Fe_*x*_N.

The small absorption area observed for the Cl_2_-treated catalyst is not caused by the removal of Fe from the catalyst, because after H_2_ treatment (without the addition of Fe), the absorption area is relatively large. This indicates that the Fe species present after the Cl_2_ treatment are not tightly bound to the carbon matrix and are not in the form of large crystallites. Such species would be undetectable by Mössbauer spectroscopy, since the recoil-free absorption requires the Fe atoms to be fixed in a solid lattice^44^.

In addition, we performed Mössbauer spectroscopy on the catalyst treated with Cl_2_ at 600 °C, and a catalyst that was first treated with Cl_2_ at 900 °C and then with H_2_ at 600 °C. Both of these treated catalysts exhibited similar activity of the as-prepared catalyst as shown in Supplementary Figs 4 and 9 and their Mössbauer spectra reveal the presence of reduced Fe species (Supplementary Figs 10 and 11; Supplementary Table 4). These results indicate that Cl_2_ treatment at 600 °C does not remove all of the reduced Fe present and therefore does not alter the activity of the catalyst. Similarly, the recovered activity of the samples treated with H_2_ at 600 °C and the presence of regenerated reduced Fe show that these species can be correlated to the changes in activity that we have observed. Since the Cl_2_ treatment at 600 °C also introduces Cl into the surface of the catalyst as demonstrated by XPS in Supplementary Fig. 5, the lack of a deactivation at 600 °C indicates that the presence of Cl on the surface of the catalyst does not cause the deactivation observed with Cl_2_ treatment at 900 °C. Cl is removed by the H_2_ treatment at 600 °C as shown in Supplementary Fig. 12, but because surface Cl does not effect the catalyst activity the simultaneous reduction of Fe species must be responsible for the reactivation.

The magnetic nature of the samples was further investigated using vibrating sample magnetometry (VSM; Supplementary Fig. 13). Superparamagnetic Fe species are present in the as-prepared and H_2_-treated catalysts, as indicated by the sigmoidal curve and lack of a ferromagnetic hysteresis. In the Cl_2_-treated catalyst, the superparamagnetic signal is diluted by the presence of paramagnetic Fe species. Supplementary Fig. 14 displays the electron paramagnetic resonance (EPR) spectra that indicate the presence of paramagnetic species in the as-prepared and Cl_2_-treated catalysts, but not in the H_2_-treated catalyst. In all EPR spectra superparamagnetic Fe species contribute a broad background signal.

### X-ray absorption spectroscopy

**Figure 4:**
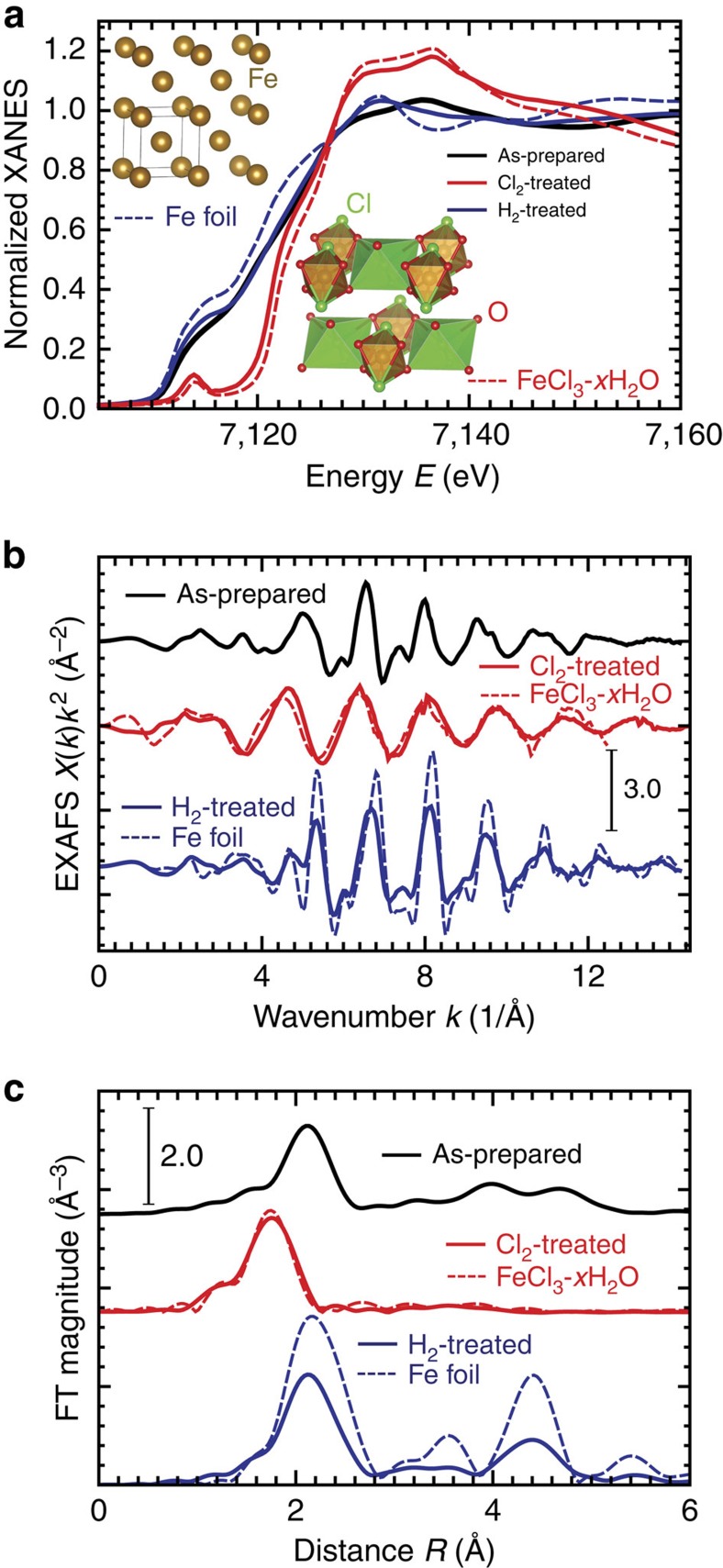
X-ray absorption spectra for as-prepared, Cl_2_-treated and H_2_-treated catalysts. XANES (**a**), EXAFS (**b**) and Fourier transforms (FTs) of EXAFS (**c**). Dashed lines show spectra for reference materials: Fe foil (bcc structure) and hydrated Fe (III) chloride (FeCl_3_·*x*H_2_O). Structures of reference materials are shown in the insets of **a**.

Fe K-edge EXAFS and XANES spectra were used to determine the local bonding environment of the Fe in each sample. Figure 4a shows the normalized XANES spectra for the catalysts studied and standards with insets displaying the structural models of the standards that are used. The spectra of the as-prepared and the H_2_-treated catalysts both show strong metallic character, resembling the spectrum of metal Fe foil. The spectrum of the Cl_2_-treated catalyst shows Fe in a higher oxidation state with a structure very similar to hydrated FeCl_3_ (FeCl_3_·*x*H_2_O)^45^. Figure 4b,c shows the corresponding Fe K-edge EXAFS data in *k*-space and after their Fourier transform to *R*-space. It is evident that in the Cl_2_-treated catalyst the interatomic distances are shorter, compared with the as-prepared and H_2_-treated catalysts, and the nearest neighbours of Fe in the Cl_2_-treated catalyst are relatively light elements, similar to those in FeCl_3_·*x*H_2_O. These conclusions follow from the lower *R*-space peak distances (Fig. 4c), and the fact that EXAFS oscillations are peaked at lower values of wavenumber *k* (Fig. 4b), respectively. In the H_2_-treated catalyst EXAFS spectra show strong similarity to Fe foil that hints on the bcc structure of the former. Lower amplitudes of EXAFS oscillations for the H_2_-treated catalyst with respect to those of metallic foil reveal reduced coordination numbers and/or enhanced structural disorder. One can thus expect that metallic Fe is in a disordered and nanostructured form in H_2_-treated catalyst.

**Figure 5:**
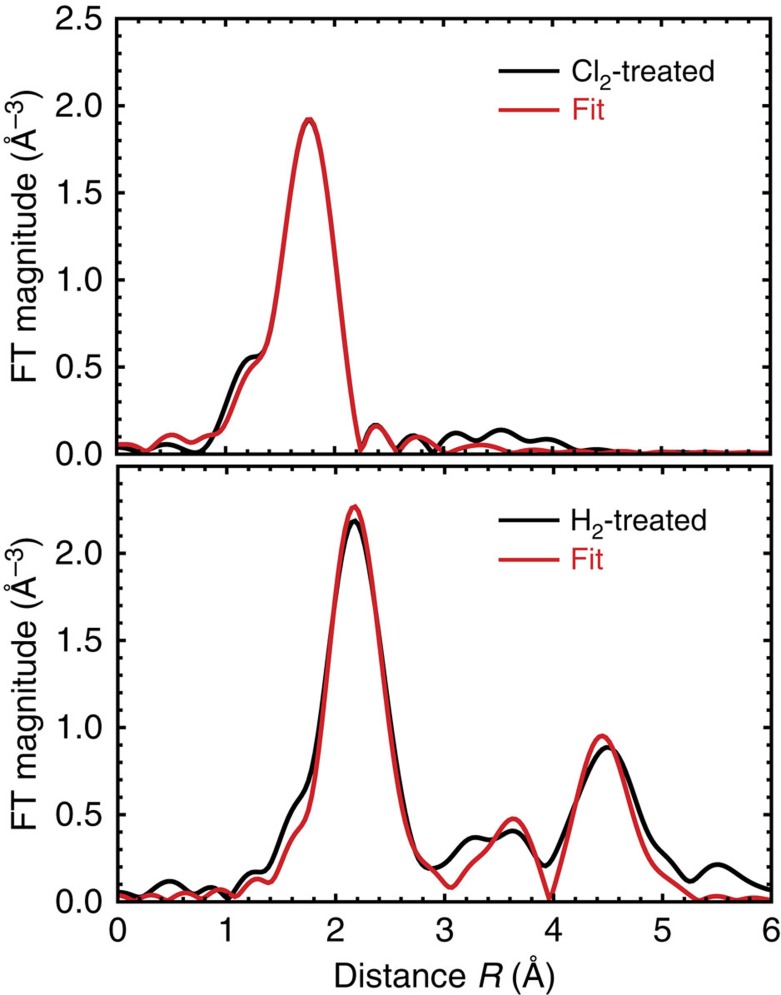
FEFFIT analysis for Cl_2_-treated and H_2_-treated catalyst material. Fourier transforms of experimental EXAFS spectra and best fit results.

Quantitative analysis of EXAFS data in the Cl_2_- and H_2_-treated catalysts was performed by non-linear least square fitting of theoretical EXAFS spectra to the data in *R*-space, using FEFFIT program^46^. Theoretical photoelectron scattering amplitudes and phases were calculated using *ab initio* FEFF8.5 code^46^,^47^. First coordination shell fitting was performed for the Cl_2_-treated catalyst, while advanced multiple-shell analysis was used for the H_2_-treated catalyst (see details in Supplementary Note 1). Data and best fits are displayed in Fig. 5. A summary of the best fit values of structural parameters are given in Table 1. For the Cl_2_-treated sample, the coordination numbers of Fe with O and Cl were found to be 2±1 and 4±1, respectively, and the Fe–Cl distance was obtained to match that of FeCl_3_˙*x*H_2_O. These values indicate the presence of hydrated FeCl_3_ and lack of metallic Fe in the sample following the Cl_2_ treatment.

For the H_2_-treated catalyst, we find that bcc model provides a good fit, in support of our preliminary conclusions from examination of the raw data. The coordination numbers for the first two nearest neighbour Fe–Fe shells for the H_2_-treated catalyst were obtained to be 5.7±0.8 and 3±2, respectively, that is, significantly smaller than the corresponding values for bulk bcc Fe (8 and 6, respectively). This indicates that the Fe is present in the nanoparticle form^48^. From the best fit values of the coordination numbers of the first few shells, the nanoparticle size can be estimated, assuming particular structure and shape^48^. Assuming the bcc-type structure and cubic shape, the particle size in the H_2_-treated sample is calculated to be ∼1.0–1.5 nm (see Supplementary Fig. 15 for details). This finding is consistent with other recent reports on Fe-based electrocatalysts^33^,^49^. The values of the mean-square relative displacement for Fe–Fe pairs were slightly larger for the H_2_-treated catalyst compared with Fe foil, indicating the presence of larger structural disorder, which is expected in small particles.

To further evaluate the size and disorder of the Fe nanoparticles in the as-prepared and H_2_-treated catalysts, we used transmission electron microscopy (TEM) and scanning TEM (STEM). Supplementary Figs 16 and 17 show TEM and STEM images of all catalysts studied, respectively. Fe nanoparticles ranging from 10 to 30 nm are observed in the as-prepared and H_2_-treated samples, while in the Cl_2_-treated sample no nanoparticles are seen, but evidence of the graphitic carbon shells previously formed around Fe nanoparticles is visible. In addition, we carried out fitting of the VSM data for the as-prepared and H_2_-treated samples using the Langevin function for superparamagnetic particles giving a third measurement of particle size (Supplementary Fig. 18; Supplementary Note 2)^33^. For the as-prepared catalyst, the average particle size from fitting was 3.22 nm, while the average particle size for the H_2_-treated catalyst was 3.80 nm. Powder X-ray diffraction indicated the presence of reduced Fe species in the as-prepared and H_2_-treated catalysts; however, the signal from the carbon support prevented further analysis of the X-ray diffraction data (Supplementary Fig. 19).

The discrepancy in the size of the Fe particles for the as-prepared and H_2_-treated catalysts as measured using TEM/STEM, EXAFS and VSM can be explained by the size ranges that each technique is able to sample and indicates a high degree of heterogeneity and, possibly, disorder among the reduced Fe species that are observed. Similar results have been obtained in a recent study using EXAFS and (S)TEM on a material with heterogeneous particle sizes, which showed that these techniques complement and do not contradict each other^50^. In addition, disagreement between TEM and VSM has been reported previously for two types of NPM ORR catalysts, possibly due to granularity in the particles observed by TEM and by the presence of disordered Fe phases within the observed particles which are non-magnetic^33^.

### X-ray photoelectron spectroscopy

**Figure 6:**
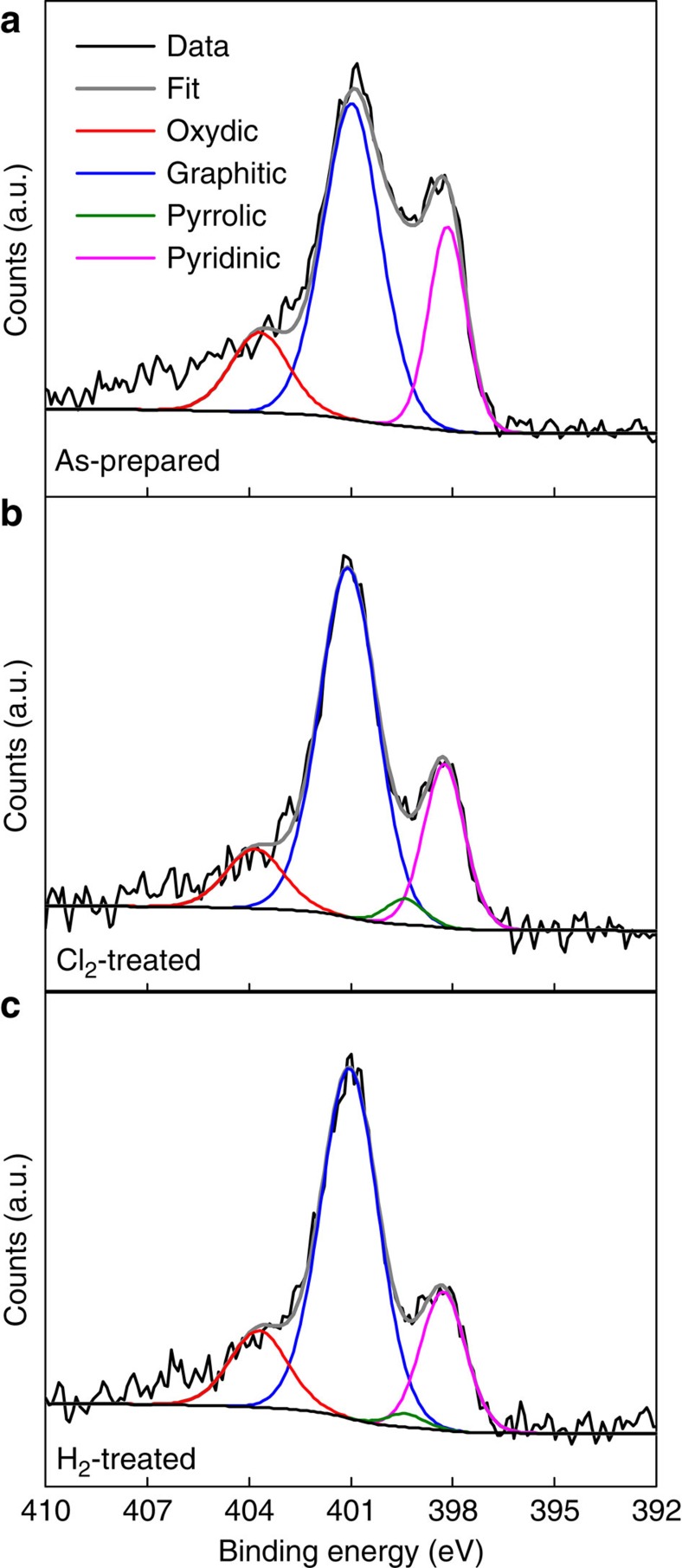
N 1 s XPS characterization of catalysts. Spectra and peak fitting of as-prepared (**a**), Cl_2_-treated (**b**) and H_2_-treated (**c**) catalysts showing the presence of oxydic (red), graphitic (blue), pyrrolic (green) and pyridinic (pink) N species.

To determine the role of N in the Fe-containing and metal-free catalysts used in our study, we carried out high-resolution XPS in the N 1 s region. In recent work on NPM and metal-free catalysts, the direct involvement of N species during ORR, especially pyridinic N and Fe–N, has been implicated^27^,^51^,^52^. The resulting spectra for the Fe-containing catalyst are shown in Fig. 6 and the spectra for the metal-free catalyst are shown in Supplementary Fig. 20. For both the Fe-containing and metal-free catalysts oxydic, graphitic, pyrrolic and/or pyridinic N species were observed as assigned from the previous literature and given in Supplementary Table 5 (refs 24, 27). Following Cl_2_ treatment and H_2_ treatment, the bands associated with pyridinic species decreased slightly, but no major changes in N speciation occurred. The fact that there were no major changes in N speciation following the Cl_2_ and H_2_ treatments suggests that the type of N species that is present does not explain any of the changes in ORR activity that we have observed. Therefore, it is likely that the active site in these catalysts does not involve N or Fe–N moieties. Graphitic N is the dominant species in all of the catalysts that we studied, which indicates that N is doped into the graphitic carbon that surrounds and protects the Fe nanoparticles. The presence of graphitic N in active NPM ORR catalysts is also observed in other work^16^,^27^,^33^.

## Discussion

**Figure 7:**
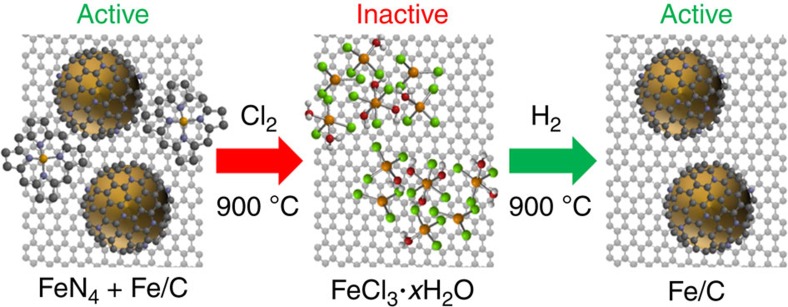
Effect of Cl_2_ and H_2_ treatments on Fe species and ORR activity. Fe species identified in each catalyst demonstrating the selective removal of FeN_4_ sites and formation of encapsulated Fe nanoparticles in the H_2_-treated catalyst.

Mössbauer and X-ray absorption spectroscopy (XAS) show that in the as-prepared catalyst a combination of FeN_4_ and reduced Fe species is present, in agreement with previous reports, which makes the direct characterization of the active species difficult^16^,^28^,^34^. Through the use of a Cl_2_ treatment, we show that the metallic Fe and FeN_4_ species are converted into dispersed FeCl_3_·*x*H_2_O that is reformed into reduced Fe species by the H_2_ treatment as shown in Fig. 7. The absence of FeN_4_ sites in the H_2_-treated catalyst indicates that these sites are not required for the observed ORR activity. To confirm that metal centred sites are not involved in the ORR, we carried out the selective poisoning experiments using KCN similar to previous work by our group^18^. Supplementary Fig. 21 shows cyclic voltammograms in the absence and presence of CN^−^, which confirm that the behaviour of the as-prepared, Cl_2_-treated and H_2_-treated catalysts is very different from that of unpyrolyzed Fe phthalocyanine. The lack of a significant poisoning effect supports our observation that FeN_4_ species are not the active species in the catalysts we studied. XPS in the N 1 s region shows oxydic, graphitic, pyrrolic and/or pyridinic N in all of the catalysts studied, with only minor changes in the relative amounts of each species, further indicating that N speciation does not play a major role in determining the ORR activity before and after the Cl_2_ and H_2_ treatments. From these results, we conclude that a reduced Fe phase, in the form of small superparamagnetic nanoparticles that are encapsulated by thin layers of N-doped carbon, can be linked to the ORR activity of the H_2_-treated catalyst. We note that although we show FeN_4_ are not required for ORR activity, FeN_4_ centres may be active in other systems^26^,^31^.

If FeN_4_ species are not the locus of ORR activity in the H_2_-treated material, the question remains as to the role of N in ORR catalyst preparation. To address this question, we omitted aniline during synthesis to make a N-free version of the as-prepared catalyst. The resultant N-free material displayed minimal ORR activity compared with the as-prepared catalyst made with aniline as shown in Supplementary Fig. 22. Interestingly, the N-free catalyst is ferromagnetic indicating the presence of larger Fe particles that are capable of forming magnetic domains as shown in Supplementary Fig. 23. Powder X-ray diffraction on the N-free catalyst shows that all of the Fe present is in the form of Fe-oxides (magnetite and haematite), which are inactive towards the ORR (Supplementary Fig. 24). Elemental analysis of the N-free catalyst shows that the material contains virtually no C or N, suggesting that the presence of the polymeric N species is required to prevent the complete removal of C during pyrolysis (Supplementary Table 6). The association of Fe with N and N with C in the catalyst during pyrolysis must play an important role in promoting the formation of the carbon-encapsulated Fe nanoparticles that are observed. The initial coordination of Fe to N should help to form the small superparamagnetic Fe particles within the carbon support. One possible explanation for this observation is that the N species function as nucleation sites for the formation of small superparamagnetic Fe particles. Another explanation is that the use of polymeric N-containing precursors helps to template and form small Fe particles during synthesis. In this way, the recent results reported utilizing extremely low Fe loading to achieve highly active Fe-based NPM catalysts could be explained by the formation of extremely small Fe nanoparticles or Fe clusters, which are contained within the carbon support and function as the active species^26^,^31^.

There are also other possible explanations for the role of N not excluded by this work. The formation of basic N sites, which tune the surface functionality, could increase the activity of the catalyst^52^. In addition, density functional theory (DFT) calculations suggest that N incorporation decreases the work function at the surface of the carbon-encapsulated nanoparticles helping to facilitate ORR^29^. It is possible that the incorporation of N may contribute to the enhanced ORR activity of NPM catalysts through one or more different effects. More work is needed to identify the specific roles of N in both the formation of catalytic sites during synthesis and possible participation of N species in the active sites during ORR.

We further characterized the catalysts before and after ORR operation to show that no new Fe or Fe–N species were formed *in situ* and that the Fe nanoparticles remain present in the material using CV, VSM and XPS (Supplementary Note 3; Supplementary Figs 25–28). These results confirm that the characterizations performed on the catalyst materials before their operation in acidic conditions for ORR remain valid while in use. Our experimental results and characterization of the catalyst materials show that reduced Fe particles encapsulated by N-doped carbon are the locus of ORR activity in the H_2_-treated catalyst. These Fe particles are disordered and nanostructured, which enhances their catalytic properties due to the increased surface to bulk ratio, and are made stable in acid by their encapsulation.

*In situ* XAS studies of other NPM ORR catalysts show the stability of the protected Fe particles from oxidation, as well as the absence of the formation of FeN_4_ structures^29^,^30^. EXAFS reports that Fe particles encapsulated by carbon stored in ambient conditions for over 2 years are not oxidized^53^. The lack of FeN_4_ sites and the encapsulation of Fe particles could possibly help to explain the puzzling observation that CO does not poison pyrolyzed NPM ORR catalysts^35^. The absence of strong sensitivity to small molecule poisons can be explained by the electronic interaction of the active species with molecular O_2_. In this case, the donation of electron density from the Fe particle to the carbon shell allows for efficient ORR catalysis. This model for shell-encapsulated Fe nanoparticles has been implicated in the hydrogen evolution reaction, where DFT suggests that electron density is donated from the Fe particle to individual atoms in the graphitic shell^49^,^54^. DFT calculations have also been used to explain the catalytic activity of encapsulated Fe particles for the ORR that are present in many NPM ORR catalysts^29^. Furthermore, recent work has shown the important role of N doping in enhancing the catalytic activity of carbon-encapsulated Fe nanoparticles^30^.

In summary, we utilized a purification process with Cl_2_ and H_2_ treatments to produce active ORR catalysts with catalytic centres of improved definition. Two clear conclusions can be drawn: (i) FeN_4_ sites are not required to generate an active ORR catalyst and (ii) reduced Fe species protected by carbon are active catalytic species for the ORR. We showed that Fe particles encapsulated by graphitic C and N, which are present in the H_2_-treated catalyst, exhibit the same ORR activity as the as-prepared catalyst leading us to identify them as the active species. Our findings reveal that synthetic methods favouring the formation of a large number of small Fe particles encapsulated by N-doped carbon may lead to the discovery of improved NPM ORR catalysts.

## Methods

### Preparation of Catalyst Material

Synthesis of the Fe-based NPM ORR catalyst was carried out following the procedure described in the previous literature^13^,^14^,^15^. Carbon black (Ketjenblack EC 300 J) was treated in 0.1 M HCl for 24 h and then in 70% HNO_3_ at 80 °C for 8 h before being filtered and dried in an oven at 80 °C overnight. In a large round bottom flask, 2.5 ml of aniline was dispersed in 500 ml of 2.0 M HCl. The mixture was cooled in an ice bath until it was <10 °C, at which point 10 g of FeCl_3_ and 5 g of ammonium peroxydisulfate were added. After stirring for 3 h, 0.4 g of the treated carbon support was added and mixed for 48 h. The suspension was evaporated at 80 °C until dry and the resulting solid material was ground using a mortar and pestle into a fine powder. The powder was placed in a tube furnace and pyrolyzed under flowing N_2_ at 900 °C for 1 h. The sample was then ground a second time and leached in 500 ml of 0.5 M H_2_SO_4_ at 80 °C for 8 h followed by a second pyrolysis under flowing N_2_ at 900 °C for 3 h. The resulting material was labelled as ‘as-prepared' catalyst.

### Cl_2_ and H_2_ treatments

Warning: Cl_2_ is highly corrosive and toxic. H_2_ is flammable and explosion may occur. For the Cl_2_ treatment the ‘as-prepared' catalyst material was placed in a tube furnace and heated under an inert Ar atmosphere to 900 °C, at which point Cl_2_ (Matheson, 99.999%) was flowed over the catalyst for 30 min. The treated sample was then allowed to cool to room temperature under Ar and labelled as ‘Cl_2_ treated'. For the H_2_ treatment, the Cl_2_-treated catalyst was heated to 900 °C under Ar, at which point a 50:50 mixture of Ar and H_2_ was flowed over the catalyst for 30 min before cooling to room temperature under Ar. The resulting material was labelled as ‘H_2_ treated'. The same treatment under pure Ar resulted in only partial recovery of catalyst activity (Supplementary Fig. 29) and significant removal of Cl (Supplementary Table 7). To study temperature effects, the same procedure was carried out using Cl_2_ at 600 °C on the as-prepared catalyst and a H_2_ treatment at 600 °C was carried out on the Cl_2_-treated (900 °C) catalyst. We note that treatment at >900 °C resulted in activity loss, even under Ar (Supplementary Fig. 30), hence treatments at >900 °C were not used.

### Electrochemical activity measurements

The ORR activity of the prepared samples was measured using a RDE and a RRDE in a three-compartment electrochemical cell. Aqueous electrolyte solutions were prepared using Milli-Q purified water (>18 MΩ cm) and HClO_4_ (70 wt%, Fisher). Solutions were sparged with either Ar or O_2_ for 30 min before each experiment. Catalyst inks were prepared by dispersing 5 mg of catalyst powder in 175 μl of EtOH and 47.5 μl of 5% Nafion solution (Sigma Aldrich) using sonication. In all tests, 5 μl of the catalyst ink was drop-cast onto a glassy carbon electrode (*A*=0.196 cm^2^) and allowed to dry under a stream of N_2_. The glassy carbon disk was polished sequentially with 0.25 and 0.05 μm diamond polish (Buehler) and sonicated in water before use. For RRDE experiments, the Pt ring was electrochemically polished before each experiment by cycling the electrode in dilute HNO_3_ solution. The ring potential was held at 1.23 V versus RHE during RRDE experiments to efficiently oxidize peroxide to oxygen. RDE and RRDE electrodes were attached to an MSRX rotator (Pine Instruments) and rotated at 1,600 r.p.m. during all experiments. Cyclic voltammetry was carried out using a CH Instruments 760 D Electrochemical Workstation (Austin, TX) at room temperature with a ‘no-leak' Ag/AgCl (3 M KCl) reference electrode (EDAQ) separated from the working electrode by a Luggin capillary. A carbon rod counter electrode was separated from the working electrode by a glass frit. All potentials were converted to RHE by measuring the open-circuit potential of a Pt wire working electrode in H_2_-saturated electrolyte immediately following catalyst testing.

### Physical characterization

Mössbauer measurements were performed on a constant acceleration spectrometer (Knox College) at 300 K. X-ray absorption spectroscopy was carried out at sector 9 beamline sector (BM) at the Advanced Photon Source at Argonne National Lab with a beam cross section of 2.6 × 0.75 mm. Samples were studied *ex situ* by pressing the catalyst powder into a pellet. All measurements were recorded in transmission mode using a double-crystal Si (111) monochromator run at 50% detuning and ion chamber detectors filled with a mixture of He/N_2_. XPS was performed using a Kratos AXIS Ultra spectrometer with a monochromatic Al Kα (1486.6 eV) X-ray source. All binding energies were referenced to graphitic carbon at 284.5 eV. VSM was performed at 300 K using the Magnetic Property Measurement System (Quantum Design) with the sample placed in a plastic capsule and inserted into a brass rod. EPR spectra were recorded on a Varian E-line 12' Century Series X-band CW spectrometer. Powder X-ray diffraction was performed using a Siemens/Bruker D5000 diffractometer with Cu K-α radiation (*λ*=0.15418, nm). ICP-OES was carried out on a PerkinElmer 2000DV ICP-OES. Sample powders were digested using 4 ml HNO_3_, 1 ml HCl and 1 ml HF in a commercial microwave digestion until the solution became clear and no solids remained present. TEM was performed on a JEOL 2010 TEM with a LaB_6_ filament at 200 kV and STEM was performed using a JEOL 2010 F STEM with a Schottky field emitter at 200 kV.

### Data Availability

The data that support the findings of this study are available from the corresponding author upon request.

## Additional information

**How to cite this article**: Varnell, J. A. *et al.* Identification of carbon-encapsulated iron nanoparticles as active species in non-precious metal oxygen reduction catalysts. *Nat. Commun.* 7:12582 doi: 10.1038/ncomms12582 (2016).

## Supplementary Material

Supplementary Information Supplementary Figures 1-30, Supplementary Tables 1-7, Supplementary Notes 1-3 and Supplementary References.

## Figures and Tables

**Table 1:** Best fit structural parameters obtained from the analysis of EXAFS data.

	***R***_**O**_ **(Å)**	***R***_**Cl**_ **(Å)**	***σ***_**O**_^**2**^ **(Å**^**2**^**)**	***σ***_**Cl**_^**2**^ **(Å**^**2**^**)**	***N***_**O**_	***N***_**Cl**_
FeCl_3_·*x*H_2_O	2.09 (5)	2.22 (1)	0.002 (3)	0.002 (3)	1.6 (9)	2
Cl_2_-treated	2.2 (1)	2.22 (2)	0.005 (3)	0.005 (3)	2 (1)	4 (1)
						
	***R***_**1**_ **(Å)**	***R***_**2**_ **(Å)**	***R***_**3**_ **(Å)**	***R***_**4**_ **(Å)**	***R***_**5**_ **(Å)**	
Iron foil	2.47 (1)	2.85 (1)	4.03 (1)	4.73 (1)	4.94 (1)	
H_2_-treated	2.48 (1)	2.87 (1)	4.05 (1)	4.75 (1)	4.97 (1)	
						
	***σ***_**1**_^**2**^ **(Å**^**2**^**)**	***σ***_**2**_^**2**^ **(Å**^**2**^**)**	***σ***_**3**_^**2**^ **(Å**^**2**^**)**	***σ***_**4**_^**2**^ **(Å**^**2**^**)**	***σ***_**5**_^**2**^ **(Å**^**2**^**)**	
Iron foil	0.004 (1)	0.005 (1)	0.007 (2)	0.010 (1)	0.004 (1)	
H_2_-treated	0.005 (1)	0.007 (5)	0.014 (6)	0.006 (4)	0.012 (4)	
						
	***N***_**1**_	***N***_**2**_	***N***_**3**_	***N***_**4**_	***N***_**5**_	
Iron foil	8	6	12	24	8	
H_2_-treated	5.7 (8)	3 (2)	12 (6)	9 (6)	3 (3)	

Interatomic distances *R*, MSRD factors *σ*^2^ and coordination numbers for the nearest coordination shells for NPM material after Cl_2_ (top) and H_2_ (bottom) treatments and for reference materials. In the parentheses the uncertainty of the last digit is given.
