# Supplementary material for: Identification of carbon-encapsulated iron nanoparticles as active species in non-precious metal oxygen reduction catalysts
Source: Nat Commun. 2016 Aug 19;7:12582. doi: 10.1038/ncomms12582 (PMC4992170; doi:10.1038/ncomms12582)
Supplement: Supplementary Information — Supplementary Figures 1-30, Supplementary Tables 1-7, Supplementary Notes 1-3 and Supplementary References. [file ncomms12582-s1.pdf]

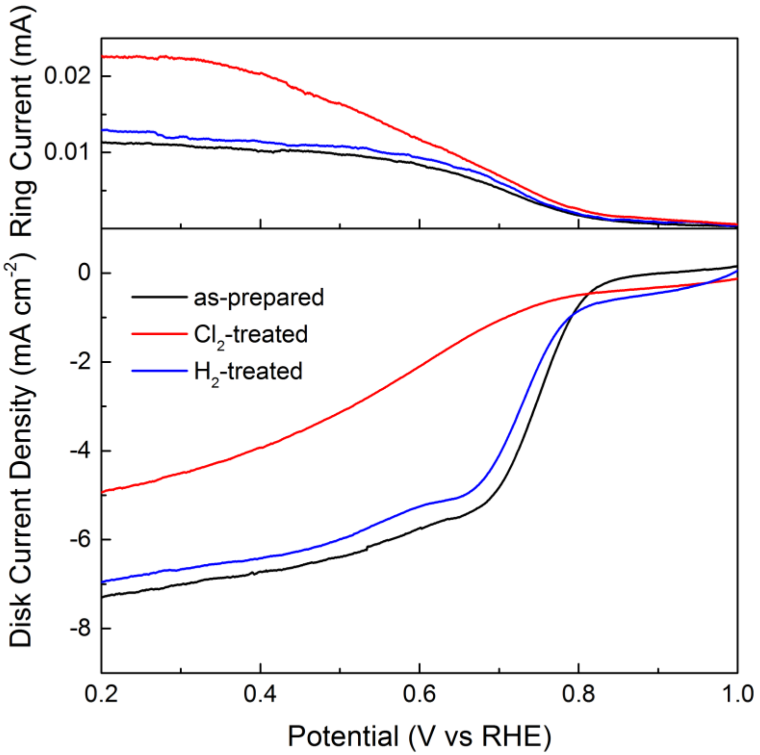

Supplementary Figure 1 | Electrochemical characterization. Linear sweep voltammograms and corresponding rotating ring disk electrode data for the as-prepared, Cl_2-treated, and H_2-treated catalysts recorded in 0.1 M $HClO_4$ with Pt ring held at 1.23 V vs RHE.

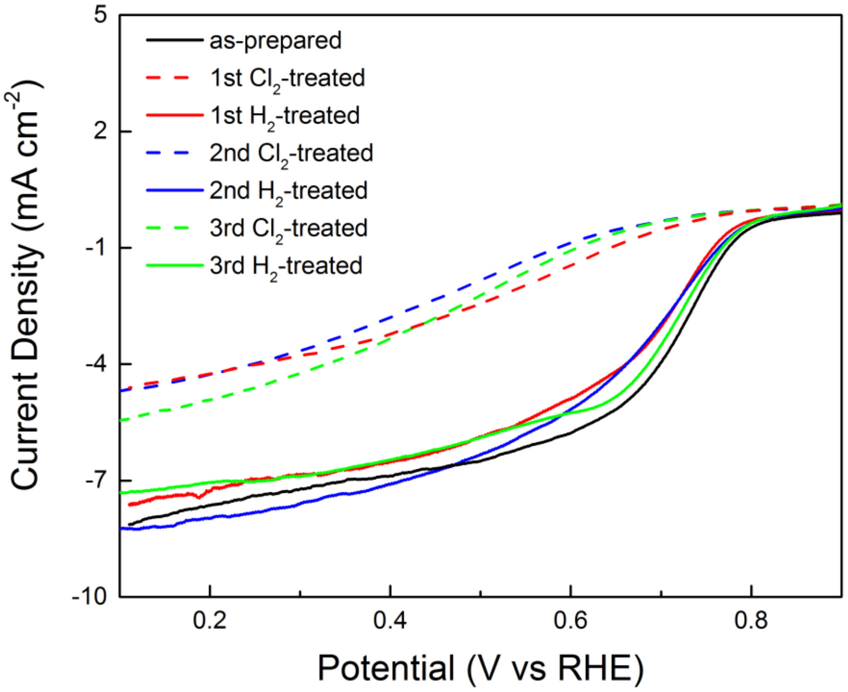

Supplementary Figure 2 | Electrochemical characterization. Cyclic voltammograms for several repeated treatments of Cl_2 and H_2 on a single batch of as-prepared catalyst in 0.1 M $HClO_4$. All Cl_2 and H_2 treatments were carried out at 900 °C for 30 minutes.

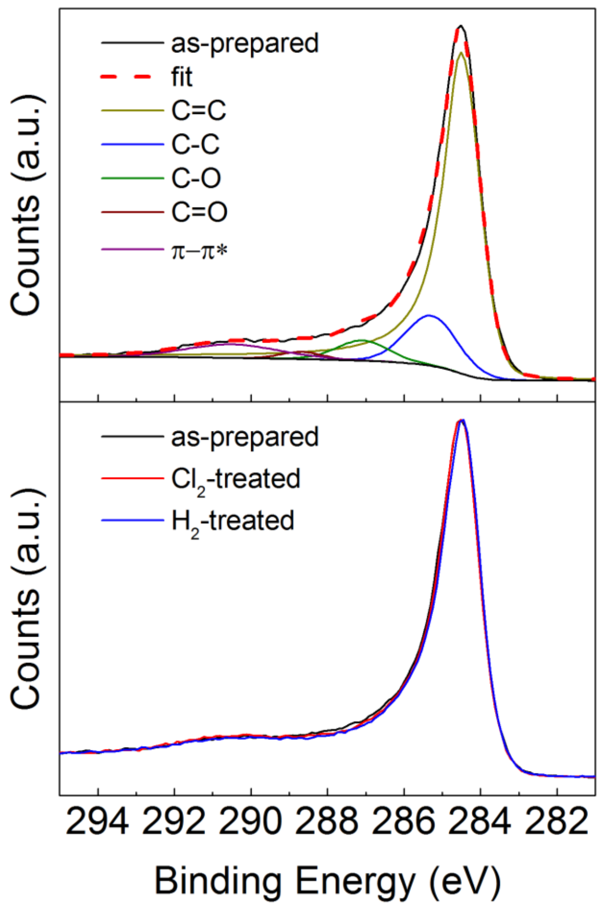

Supplementary Figure 3 | Surface characterization. C 1s XPS spectra for Fe catalysts with peak fitting for the as-prepared catalyst and overlay of intensity normalized spectra for the as-prepared, Cl_2-treated, and H_2-treated catalysts. The main signal from the carbon black was fitted to the lineshape of graphitic sp^2-type carbon.

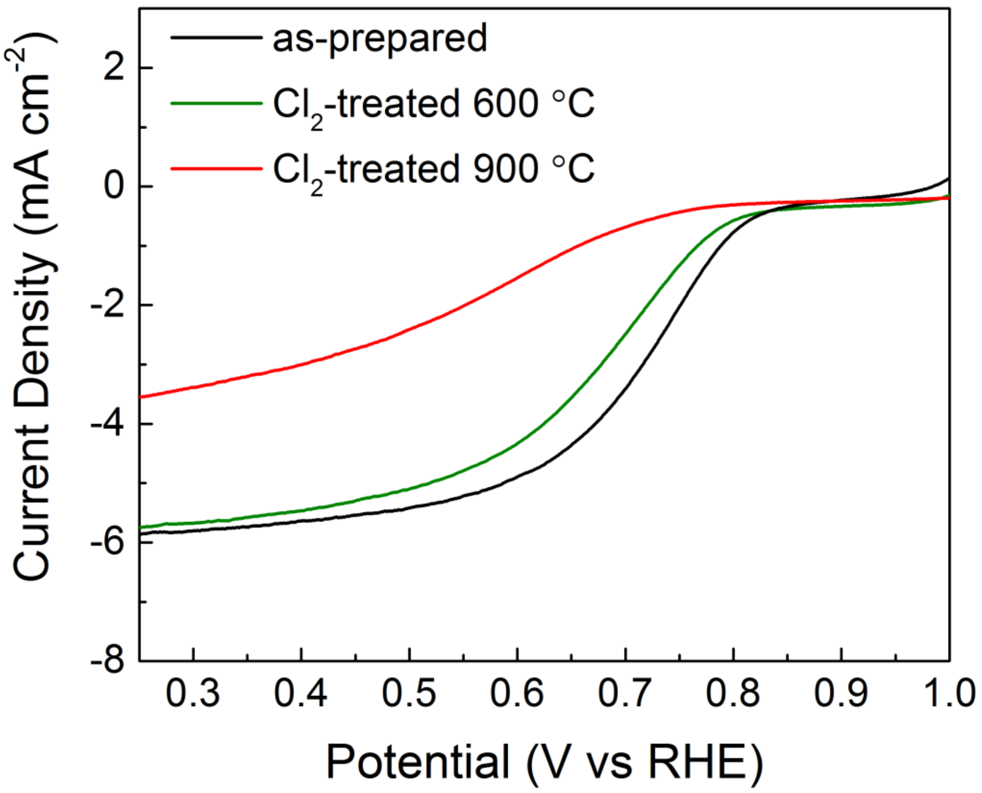

Supplementary Figure 4 | Electrochemical characterization. Cyclic voltammograms showing the ORR activity of the as-prepared catalyst before and after 600 °C and 900 °C Cl_2 treatment recorded in 0.1 M $HClO_4$.

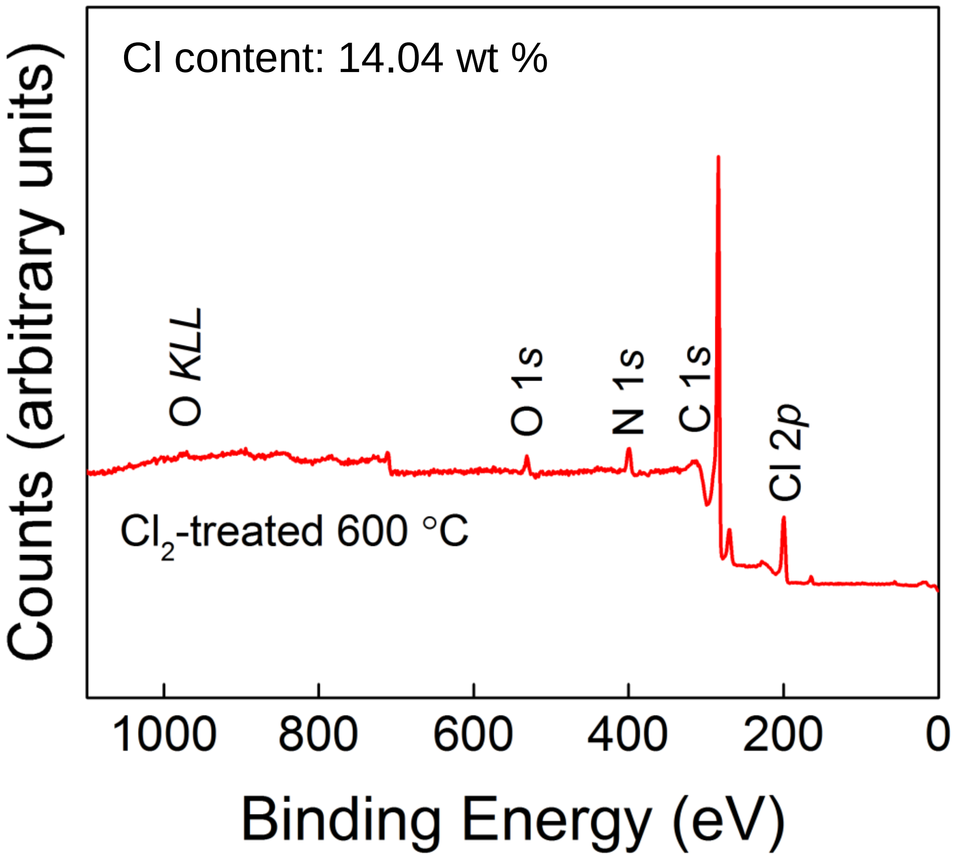

Supplementary Figure 5 | Surface characterization. XPS survey spectrum of 600 °C Cl_2 treatment. The Cl content was determined from elemental analysis.

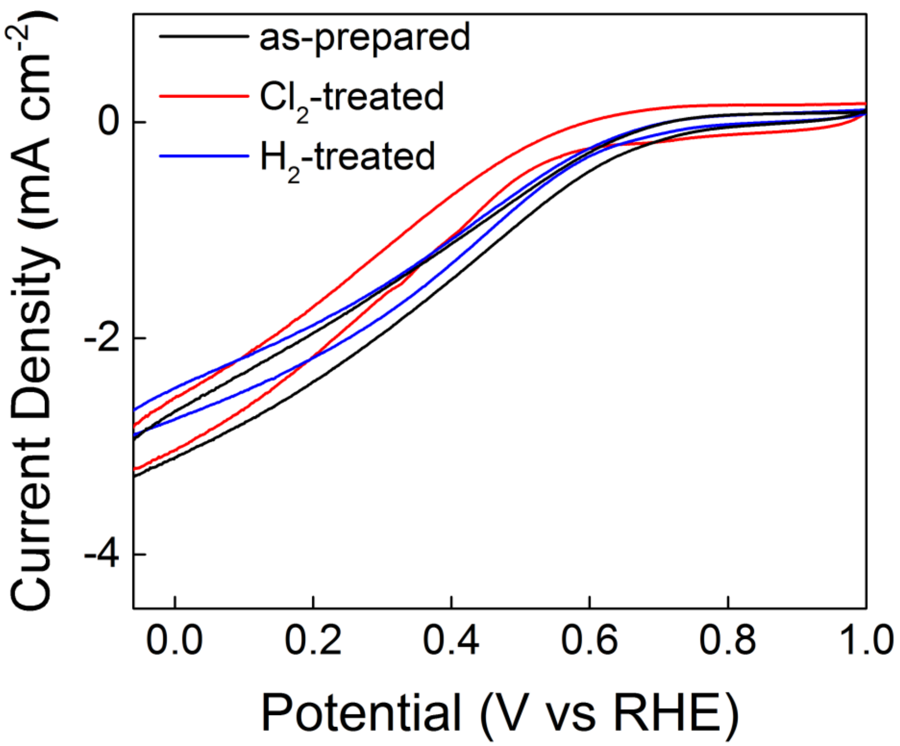

Supplementary Figure 6 | Electrochemical characterization. Cyclic voltammograms of ORR on metal-free as-prepared, Cl_2-treated, and H_2-treated catalysts in 0.1 M $HClO_4$.

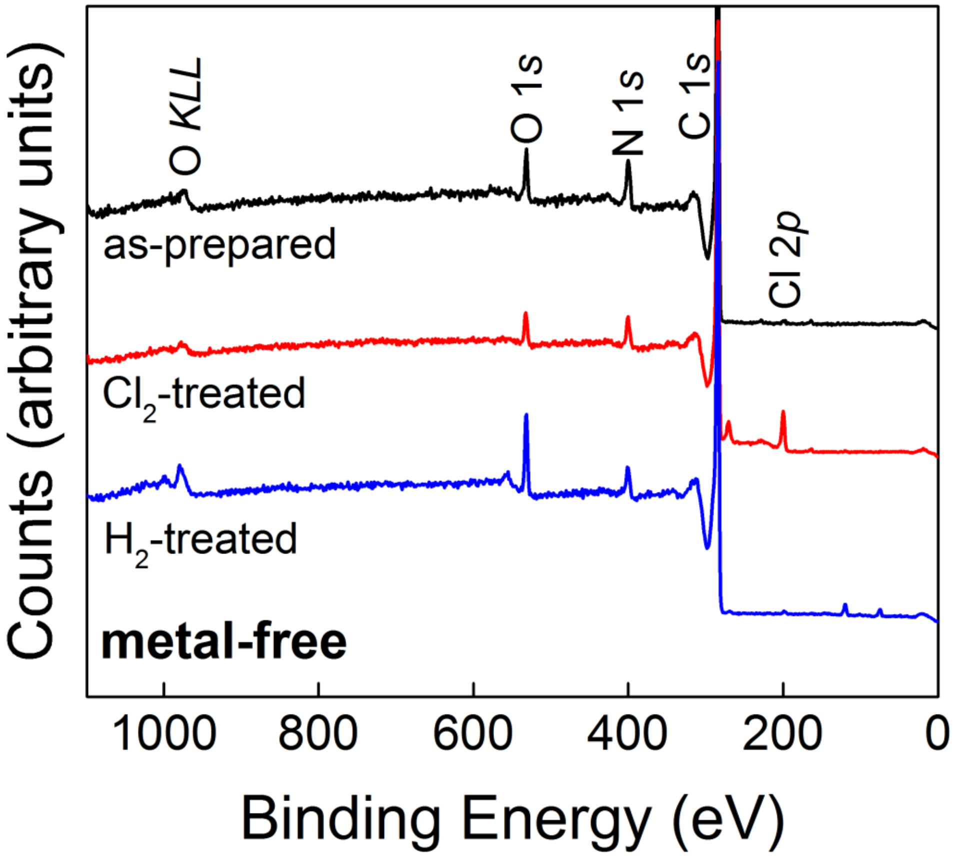

Supplementary Figure 7 | Surface characterization. XPS survey spectra for metal-free catalysts studied in this work.

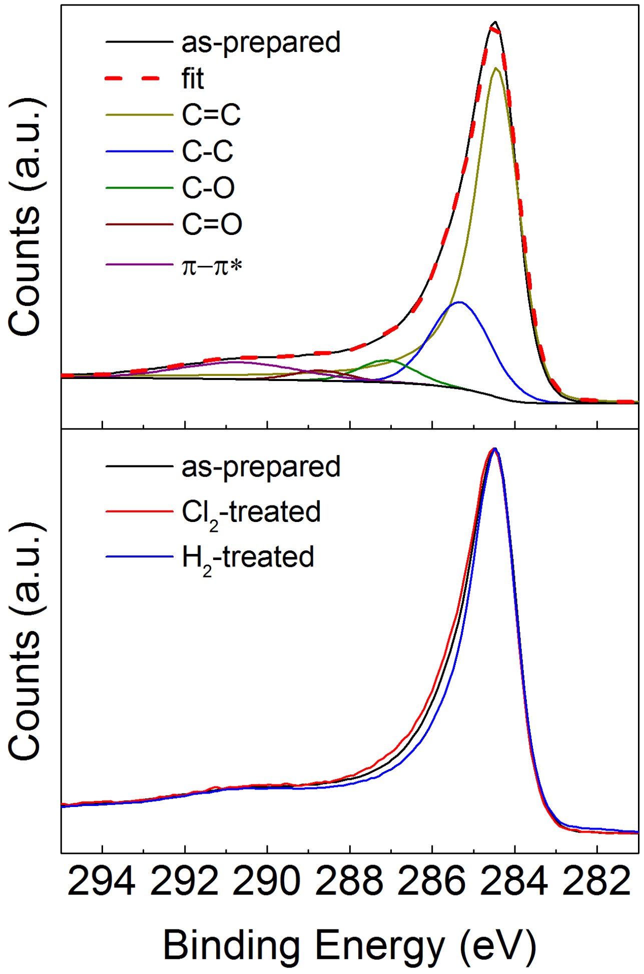

Supplementary Figure 8 | Surface characterization. C 1s XPS spectra for metal-free catalysts with peak fitting for the as-prepared catalyst and overlay of intensity normalized spectra for the as-prepared, Cl_2-treated, and H_2-treated catalysts. The carbon black was fitted to the lineshape of graphitic sp^2-type carbon.

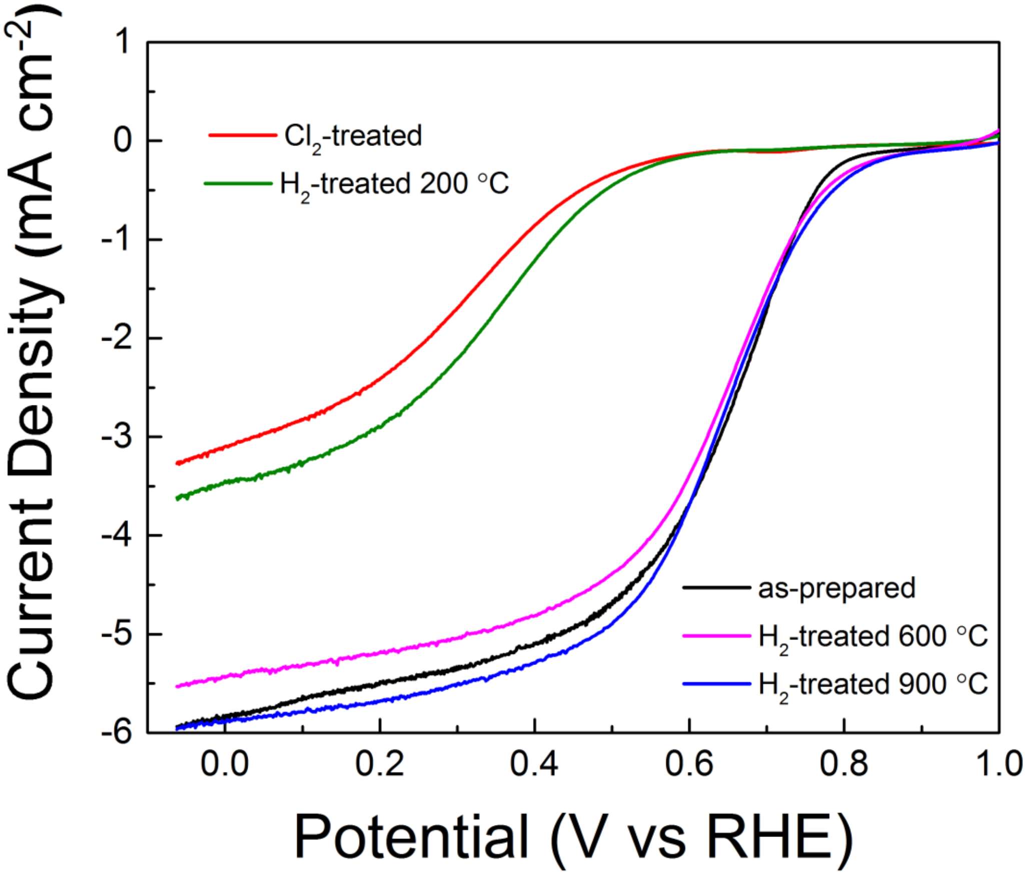

Supplementary Figure 9 | Electrochemical characterization. Cyclic voltammograms of catalyst treated with Cl_2 at 900 °C followed by treatment with H_2 at increasing temperatures.

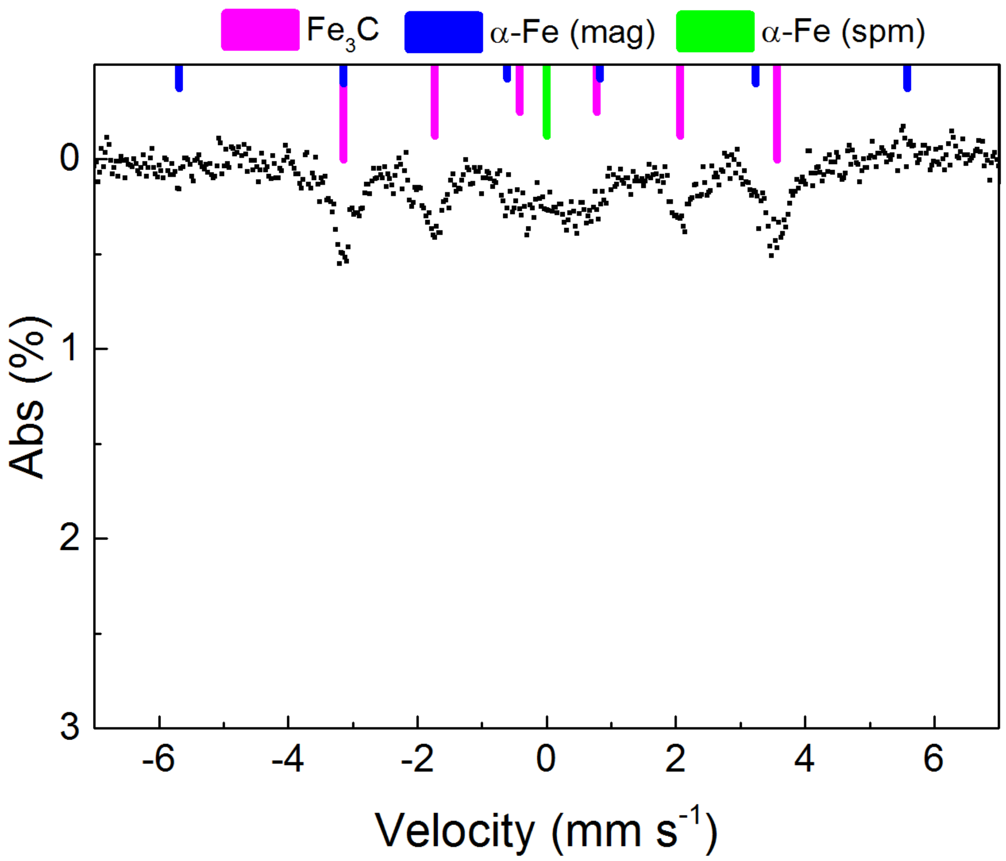

Supplementary Figure 10 | Characterization of Fe species. Mössbauer spectrum of sample treated with Cl_2 at 600 °C for 30 minutes with peak locations for reduced Fe species: Fe_3C, α-Fe (mag) and α-Fe (spm). Treatment with Cl_2 at 600 °C does not remove all reduced Fe species present in the as-prepared catalyst. The smaller absorption area signifies that only the exposed Fe is removed. We note that similar results treating with Cl_2 at 650 °C have been previously observed.[1]

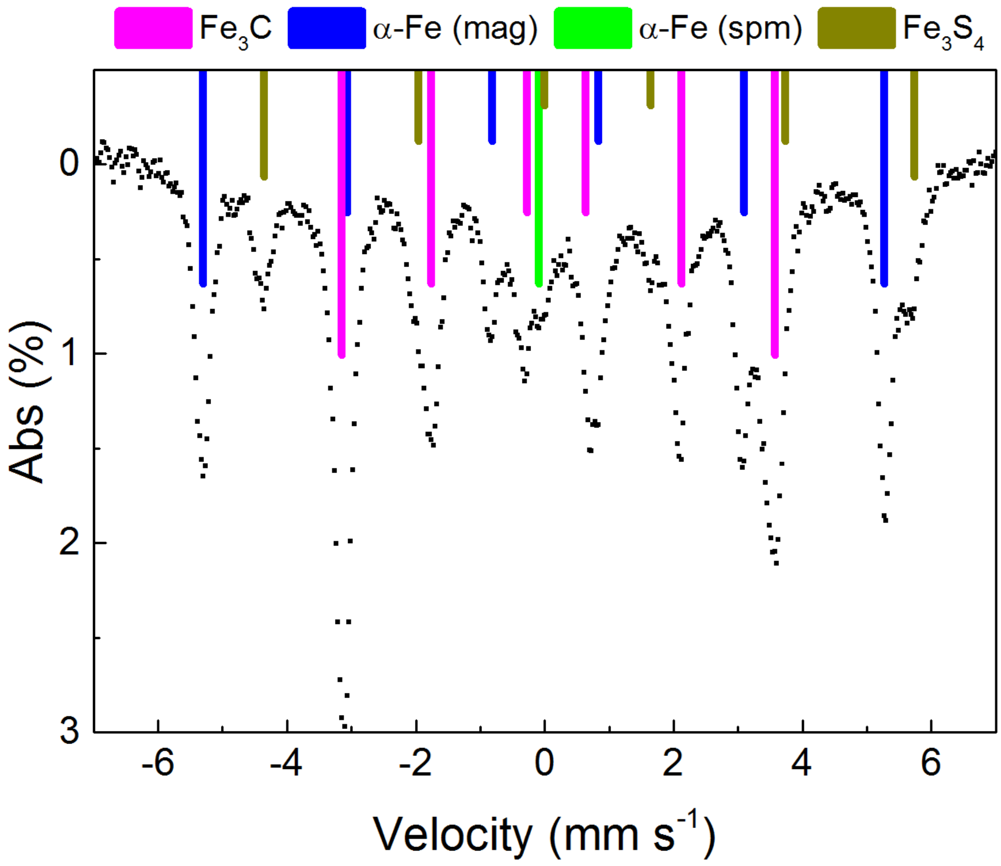

Supplementary Figure 11 | Characterization of Fe species. Mössbauer spectrum of sample treated with H_2 at 600 °C for 30 minutes following Cl_2 treatment at 900 °C with peak locations for reduced Fe species: Fe_3C, α-Fe (mag) and α-Fe (spm). Additionally, a new species identified as Fe_3S_4 is present with fitting parameters given in **Supplementary Table 4** which has been reported in previous literture.[2] It is evident that the presence of this Fe_3S_4 has no effect on catalyst activity. Treatment with H_2 at 600 °C effectively reduces the Fe present in the Cl_2-treated catalyst to reform the reduced Fe species present in the as-prepared catalyst and catalyst treated with H_2 at 900 °C.

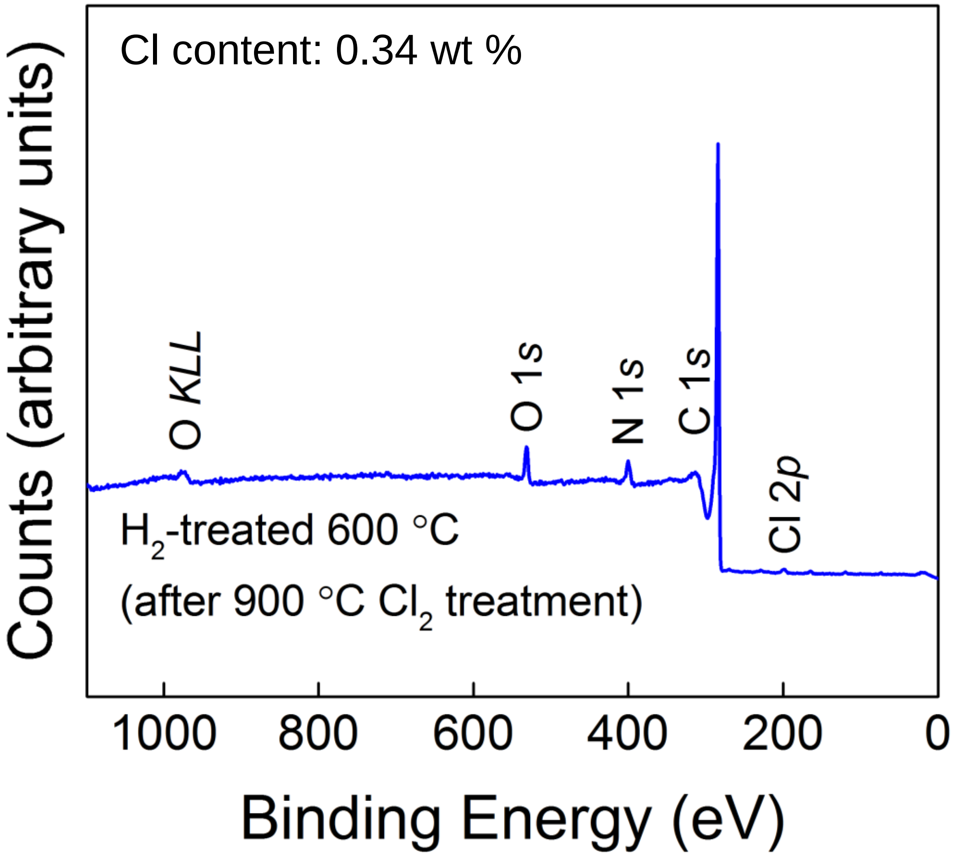

Supplementary Figure 12 | Surface characterization. XPS survey spectrum for sample treated with H_2 at 600 °C. The Cl content was determined from elemememental analysis.

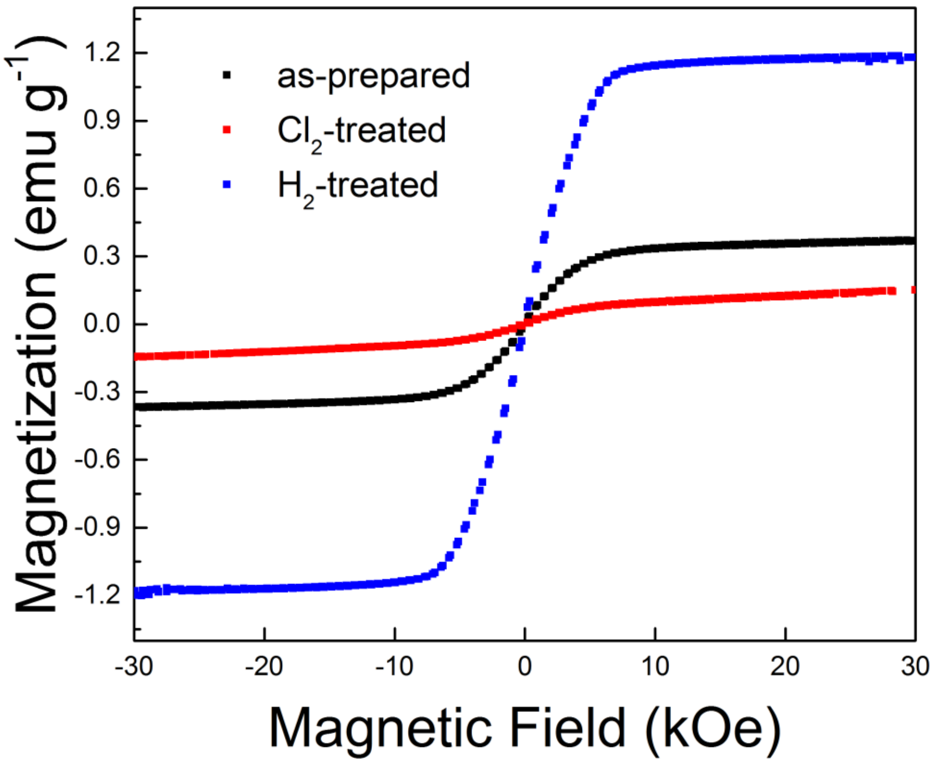

Supplementary Figure 13 | Magnetic property characterization. Vibrating sample magnetometry data at 300 K showing the magnetization of the catalyst materials. A sigmoidal shape and lack of central hysteresis indicates the presence of superparamagnetic species. A diagonal shape indicates the presence of paramagnetic species.

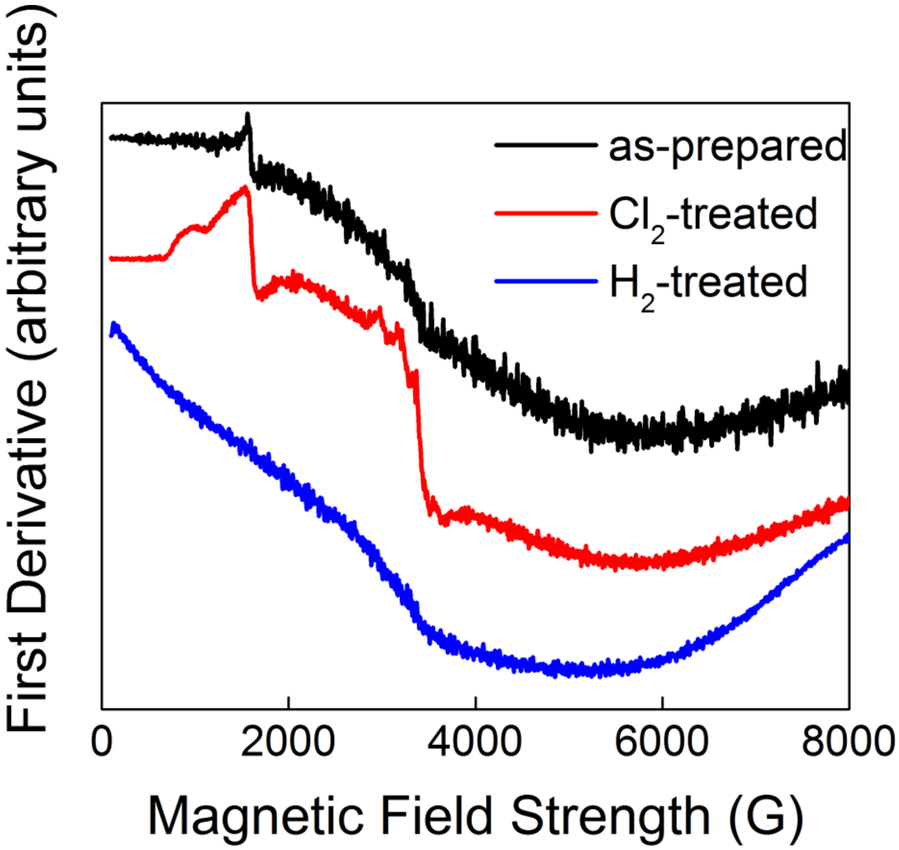

Supplementary Figure 14 | Magnetic property characterization. X-band EPR spectra recorded at 77 K for catalysts. The features at low field strength indicate the presence of paramagnetic species. The broad background indicates the presence of superparamagnetic or ferromagnetic particles. Microwave frequencies of 9.2782 GHz, 9.2848 GHz, and 9.2818 GHz were used for the as-prepared, Cl_2-treated, and H_2-treated respectively.

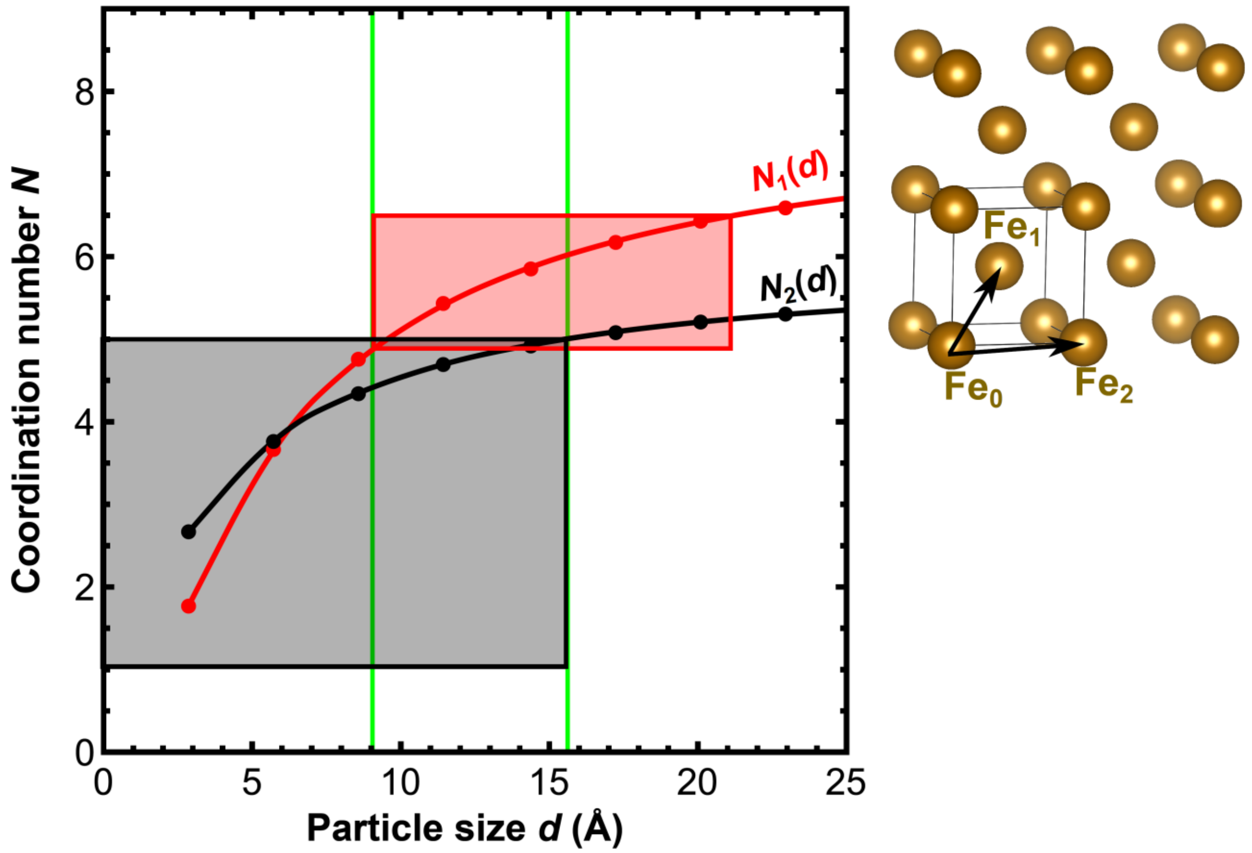

Supplementary Figure 15 | Fe particle size determination from EXAFS. Red and black dots and solid lines show calculated dependencies of coordination numbers $N_1(d)$ and $N_2(d)$ on particle size d for cubic particles with bcc-type Fe structure (see schematic picture on the right). Vertical positions and heights of filled rectangles correspond to the best fit values for coordination numbers N_1 (red) and N_2 (black) and their uncertainties for H_2-treated material. The area between two vertical green lines shows the range of particle sizes, consistent with the values for N_1 and N_2, obtained from fit.

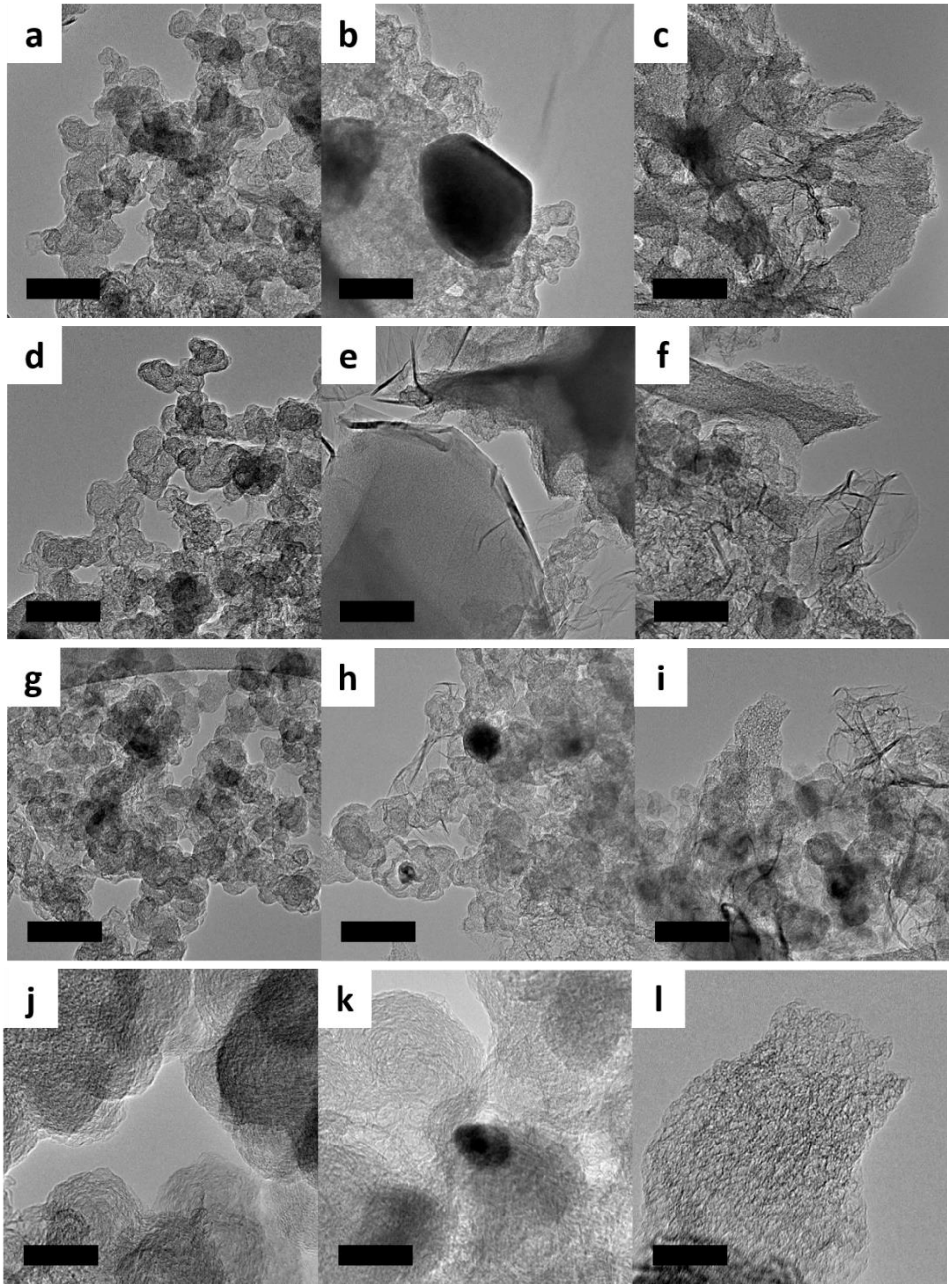

Supplementary Figure 16 | Physical characterization. Representative TEM images of the as-prepared (a-c), Cl_2-treated (d-f), and H_2-treated (g-l) catalysts. All catalysts examined show the presence of the carbon support particles c.a. 50 nm in diameter and amorphous carbon regions. The as-prepared and H_2-treated catalysts contain carbon encapsulated Fe nanoparticles (b, h, k) while the Cl_2-treated catalyst shows evidence of hollow/fractured layered carbon regions (e) indicating Fe nanoparticle removal. Scale bars (a-i) 100 nm (j-l) 20 nm.

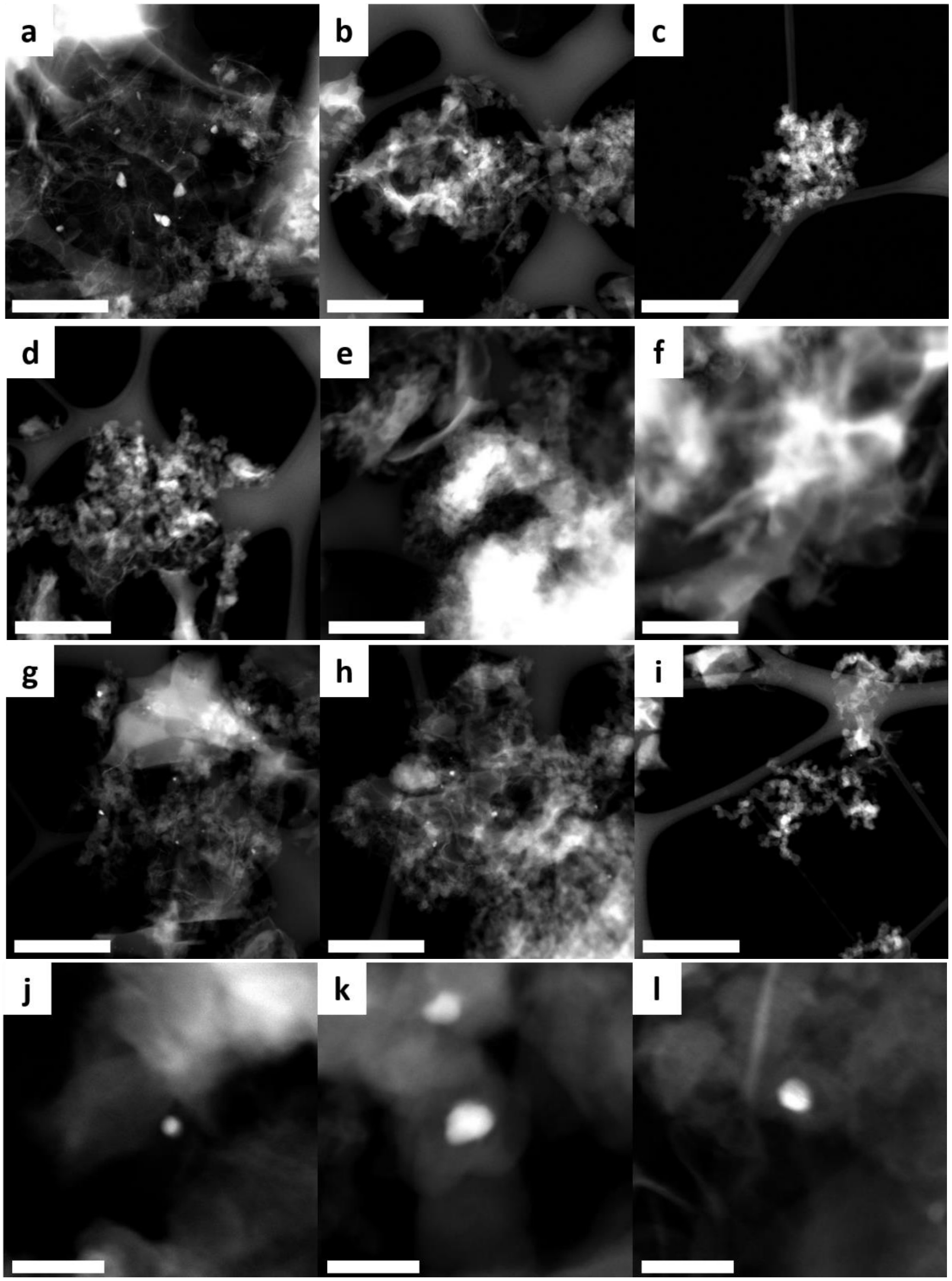

Supplementary Figure 17 | Physical characterization. Representative STEM images of the as-prepared (a-c), Cl_2-treated (d-f), and H_2-treated (g-l) catalysts. In the as-prepared and H_2-treated catalysts the presence of Fe nanoparticles is observed with the particle size in the H_2-treated catalyst around 5-25 nm. In the Cl_2-treated catalyst the presence of brighter regions caused by stronger diffraction is observed, possibly from $FeCl_3$ regions. Scale bars (a-i) 500 nm (j-l) 50 nm.

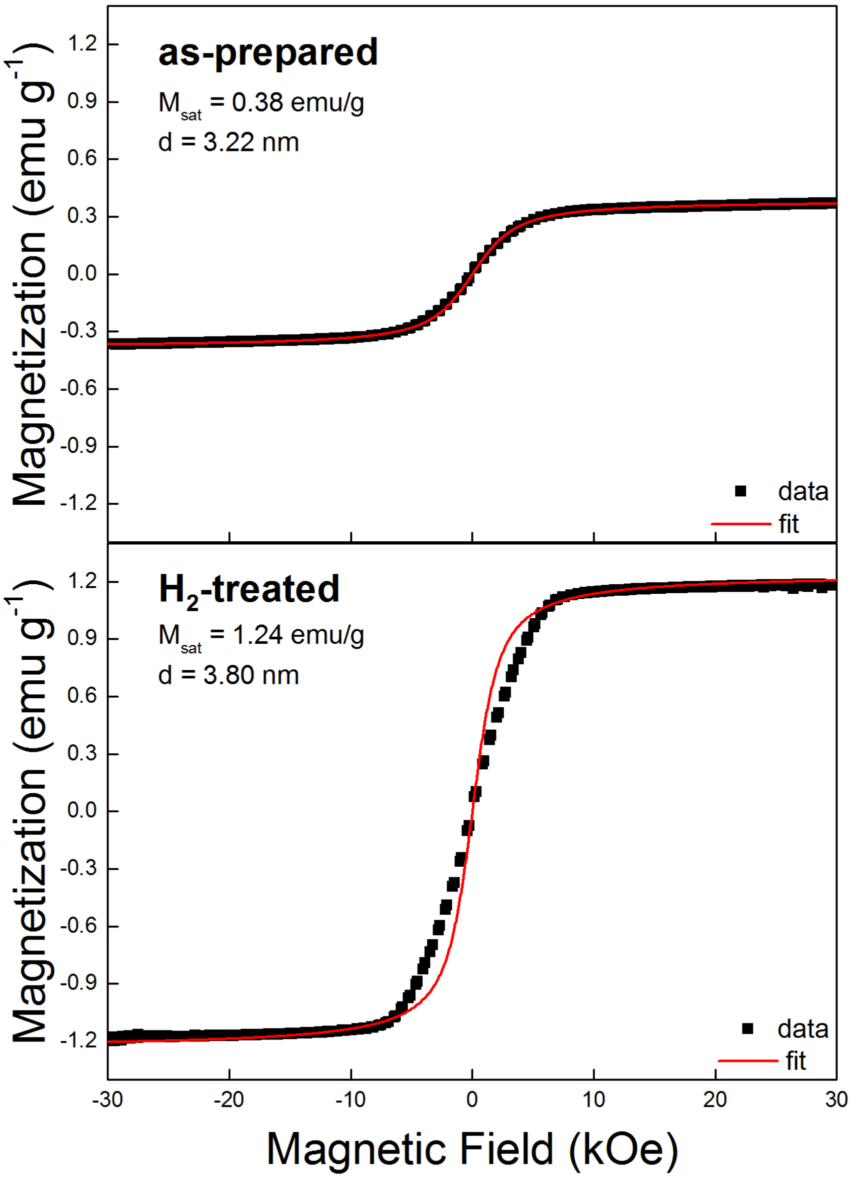

Supplementary Figure 18 | Particle size determination from VSM. VSM fitting for the as-prepared and H_2-treated catalyst materials.

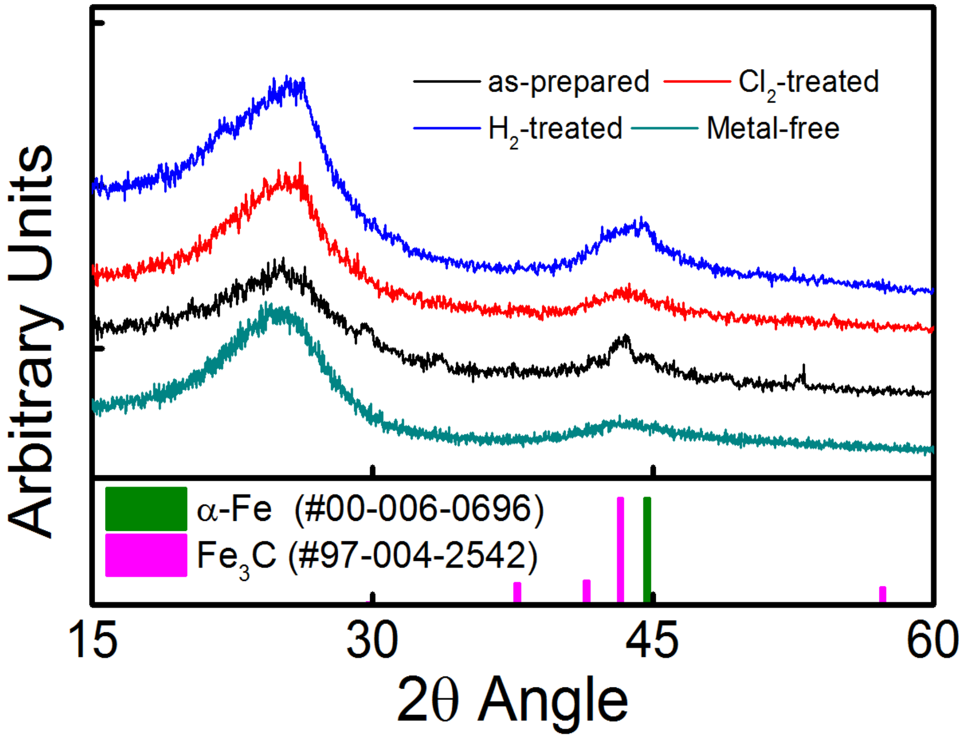

Supplementary Figure 19 | Physical characterization. Powder XRD of as-prepared and treated Fe-containing catalysts and as-prepared metal-free catalyst. Strong signal from carbon support is observed with small peaks from disordered Fe species observed in the as-prepared and H_2-treated catalysts.

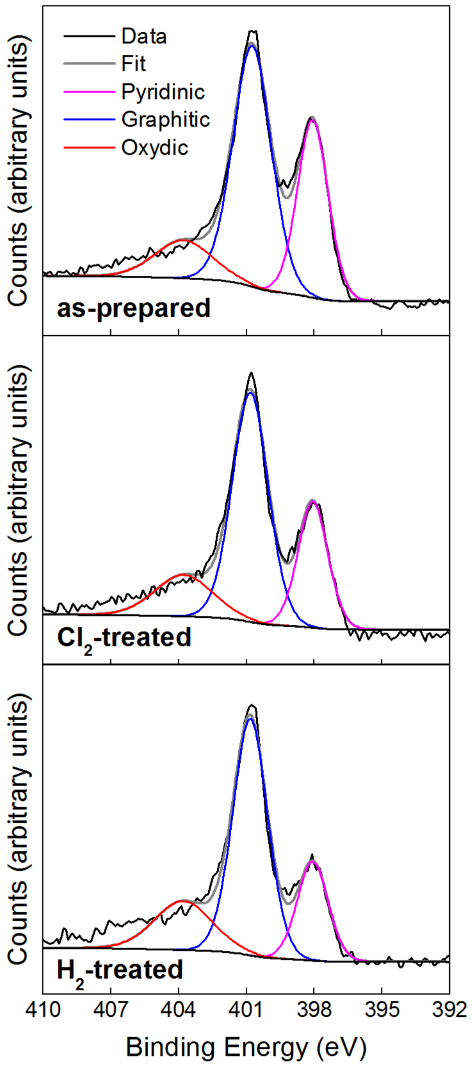

Supplementary Figure 20 | Surface characterization. N 1s XPS spectra of as-prepared, Cl_2-treated, and H_2-treated metal-free catalysts.

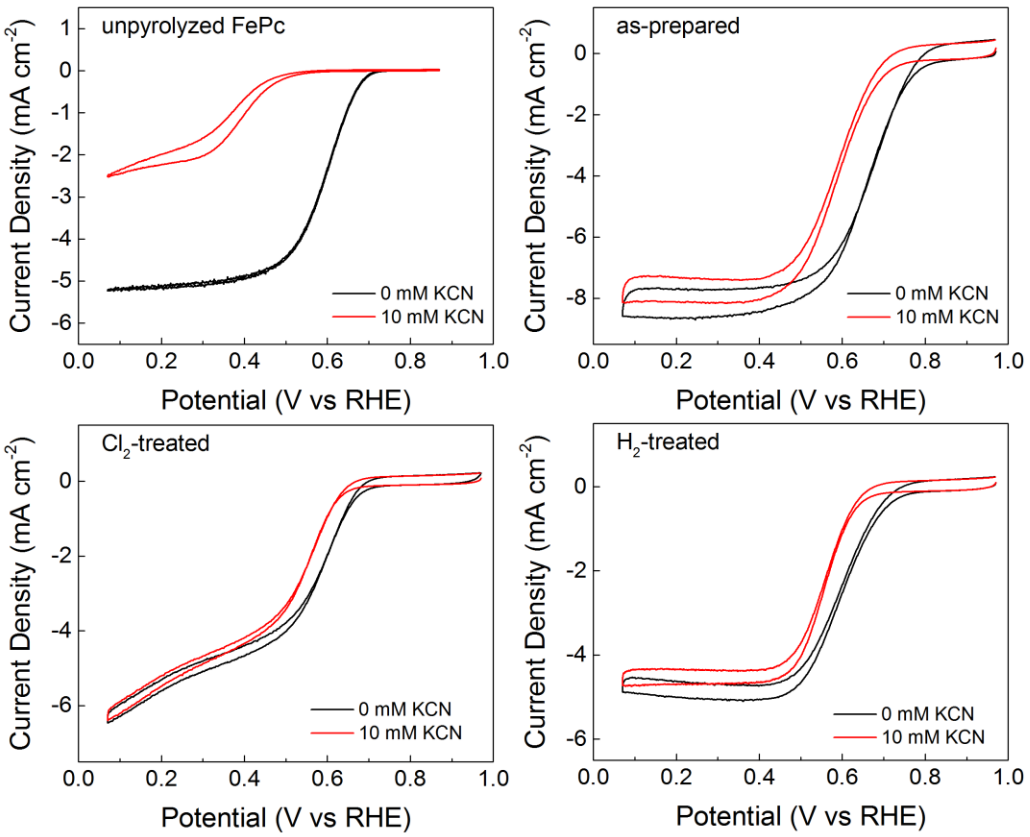

Supplementary Figure 21 | Electrochemical characterization. Cyclic voltammograms demonstrating the poisoning of ORR activity for all catalysts with 10 mM KCN in 0.1 M NaOH.

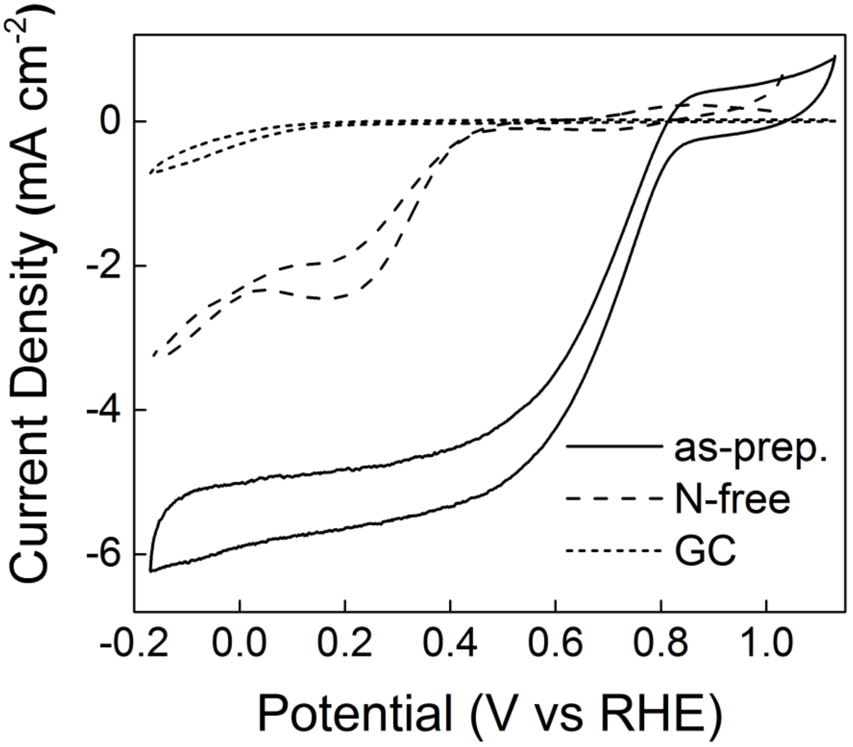

Supplementary Figure 22 | Electrochemical characterization. Cyclic voltammograms of ORR on as-prepared catalyst, N–free catalyst, and glassy carbon in 0.1 M $HClO_4$.

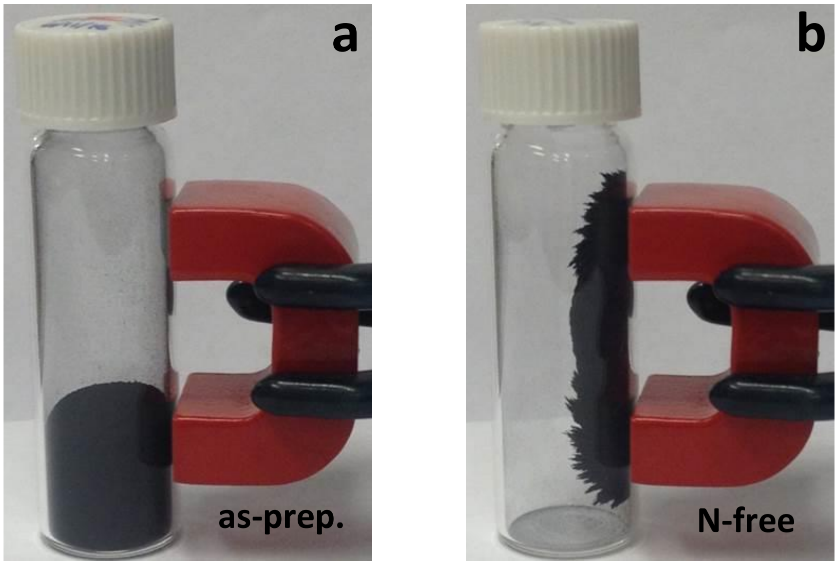

Supplementary Figure 23 | Magnetic property characterization. As-prepared (a) and N-free (b) catalysts in the presence of a magnetic field. The strong magnetic property of the N-free catalyst indicates that it is composed of material capable of forming large magnetic domains. In the as-prepared catalyst, the absence of magnetic domains indicates that it contains small, reduced Fe particles encapsulated within the carbon.

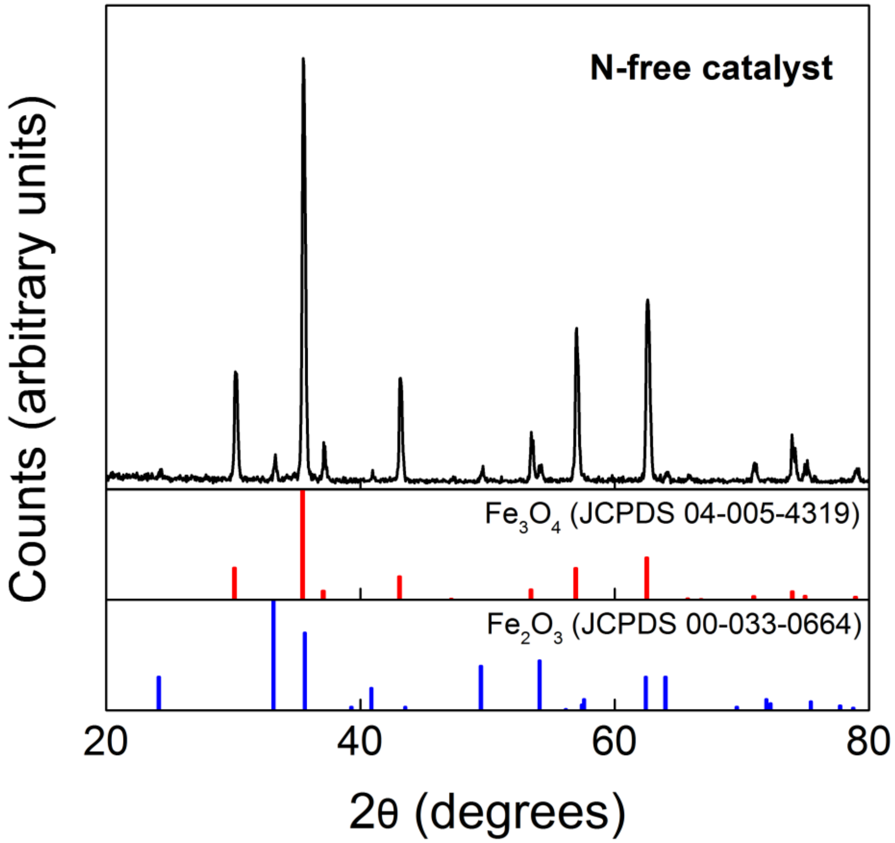

Supplementary Figure 24 | Physical characterization. Powder XRD of N-free catalyst. Diffraction bands are assigned to magnetite (Fe_3O_4) and hematite (Fe_2O_3). Average crystallite size is 49.7 ± 1.6 nm using FWHM.

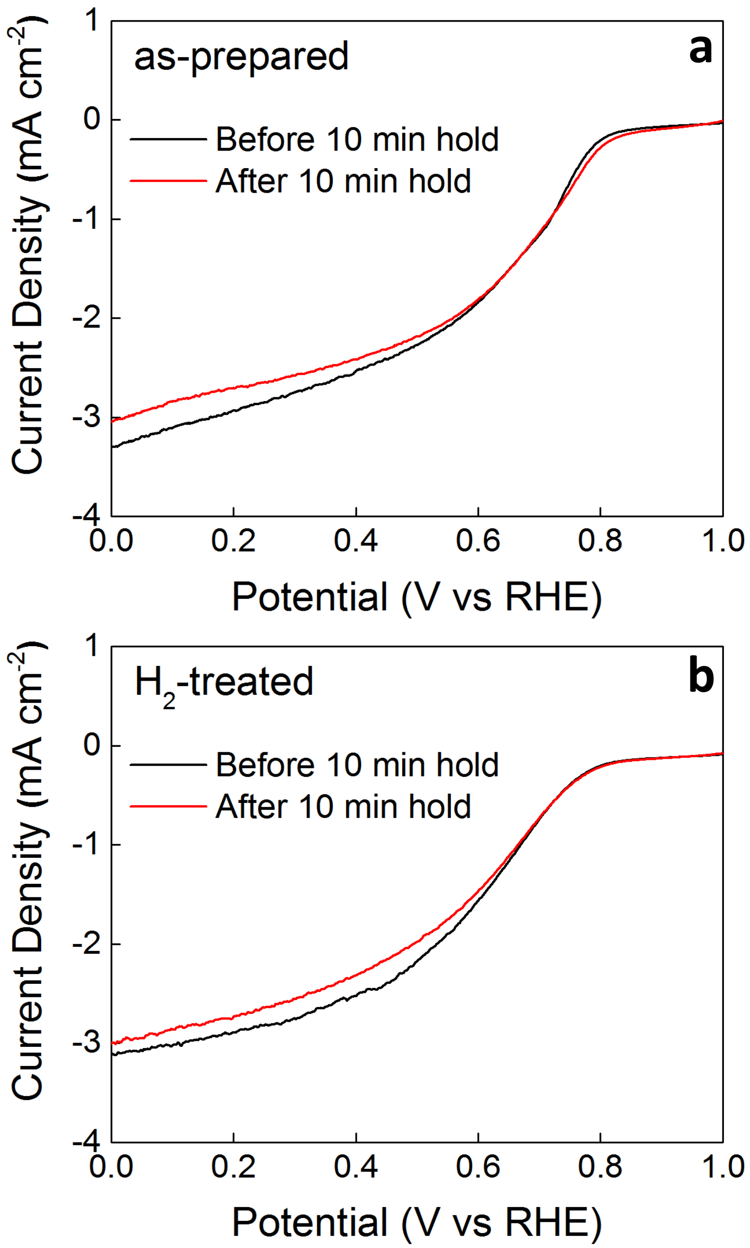

Supplementary Figure 25 | Electrochemical characterization. Cyclic voltammograms of ORR on as-prepared (a) and H_2-treated (b) catalysts before and after a potential hold at 0.2 V vs RHE in 0.1 M $HClO_4$. Results indicate that ORR activity is not significantly altered during and following operation at oxygen reducing potential. A small decrease in current was observed due to the possible loss of catalyst material or a small number of active species while rotating.

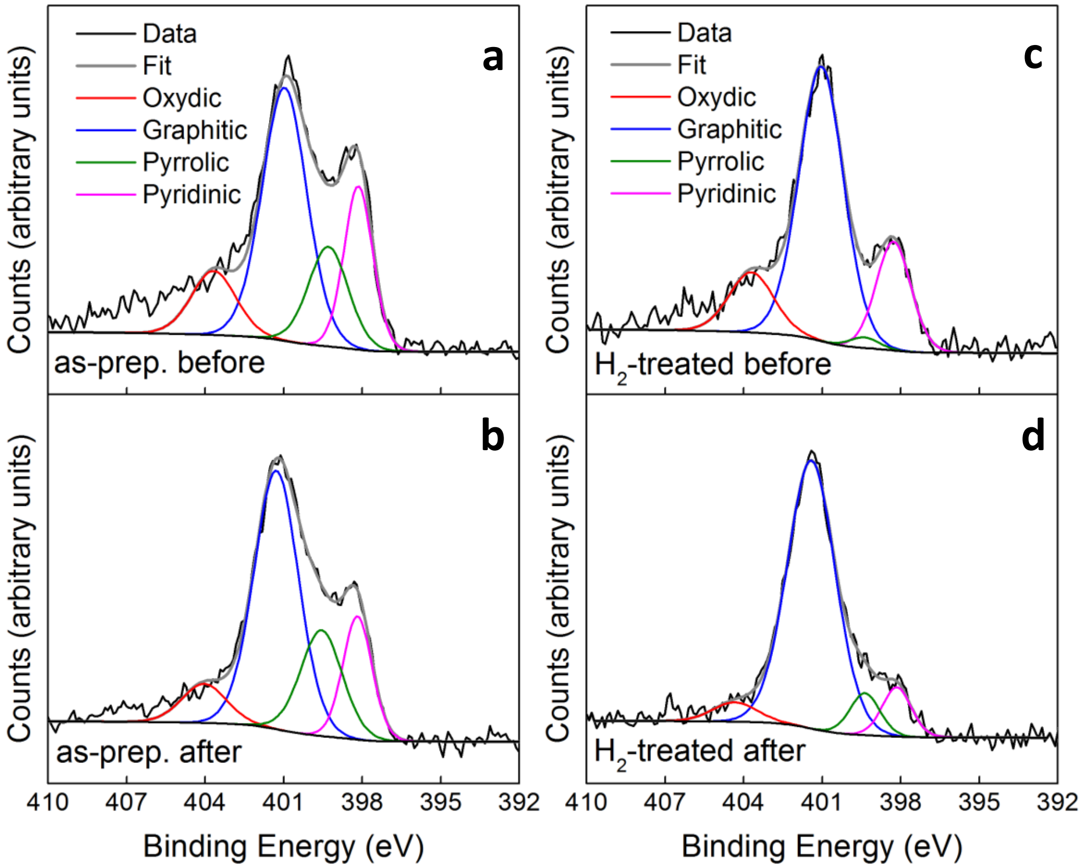

Supplementary Figure 26 | Surface characterization. N 1s spectra of the as-prepared catalyst (left) and H_2-treated catalyst (right) before (a, c) and after (b, d) a potential hold at 0.2 V versus RHE for 10 minutes in 0.1 M $HClO_4$. Changes in the pyrrolic and pyridinic bands indicate that these species do not actively participate in the ORR as no significant changes in activity were observed during operation using cyclic voltammetry.

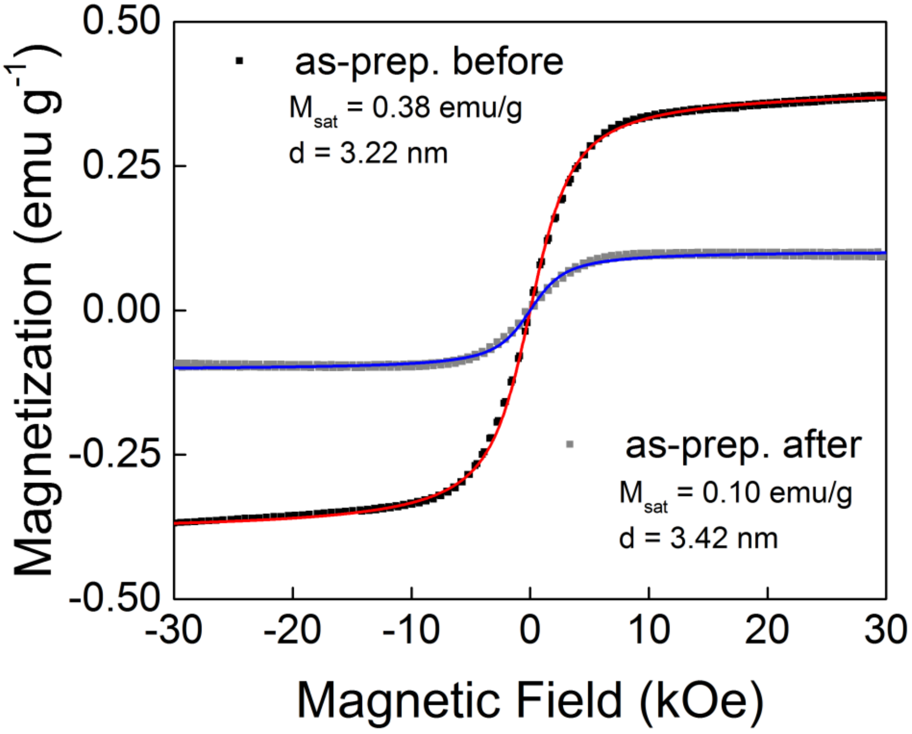

Supplementary Figure 27 | Magnetic property characterization and particle size determination from VSM. VSM data for the as-prepared catalyst before and after a potential hold at 0.2 V versus RHE for 10 minutes in 0.1 M $HClO_4$. The decrease in magnetization observed results from the dissolution of metallic species at the surface which are inactive for ORR. After operation the sigmoidal signal of superparamagnetic species is still present indicating that some particles are encapsulated and protected by carbon. Data was fitted using the Langevin equation for superparamagnetic matrials to give particle size.

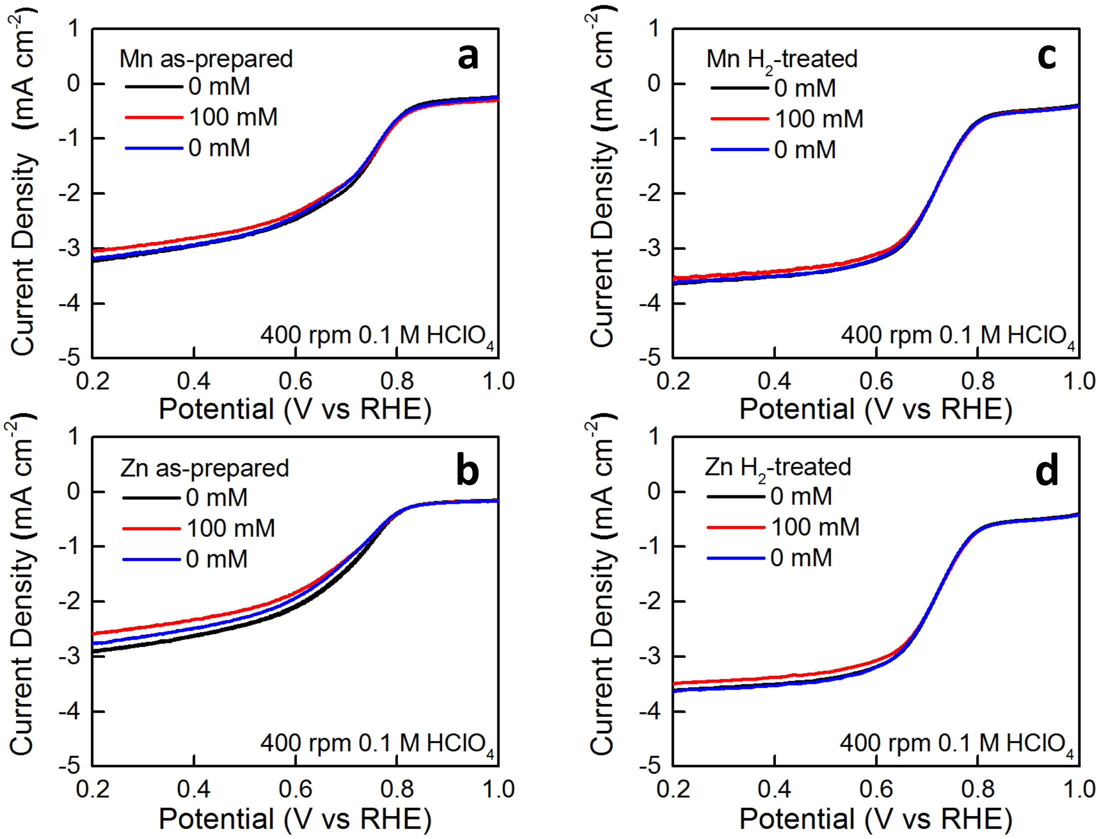

Supplementary Figure 28 | Electrochemical characterization. Linear sweep voltammograms of ORR on as-prepared (a-b) and H_2-treated catalysts (c-d) in 0.1 M $HClO_4$ before and after addition of 100 mM $ZnClO_4$ or $MnClO_4$ and after rinsing and introducing fresh electrolyte absent Zn and Mn. In order to eliminate the possibility of a new active form of the catalyst being formed upon introduction of the catalyst to the electrolyte ORR inactive metals were introduced into solution. The high concentration of these metal ions should prevent putative dissolved Fe from coordinating to the catalyst surface, perhaps at pyridinic N sites. This result, in addition to the evidence that FeN_4 sites are not present in the H_2-treated catalyst, proves that Fe species formed during operation cannot be the source of ORR activity in the catalysts studied. Small changes in the diffusion limited current were observed due to the decrease in solubility of oxygen in solutions with high salt concentration and from the possible loss of catalyst material or a small number of active species while rotating.[3]

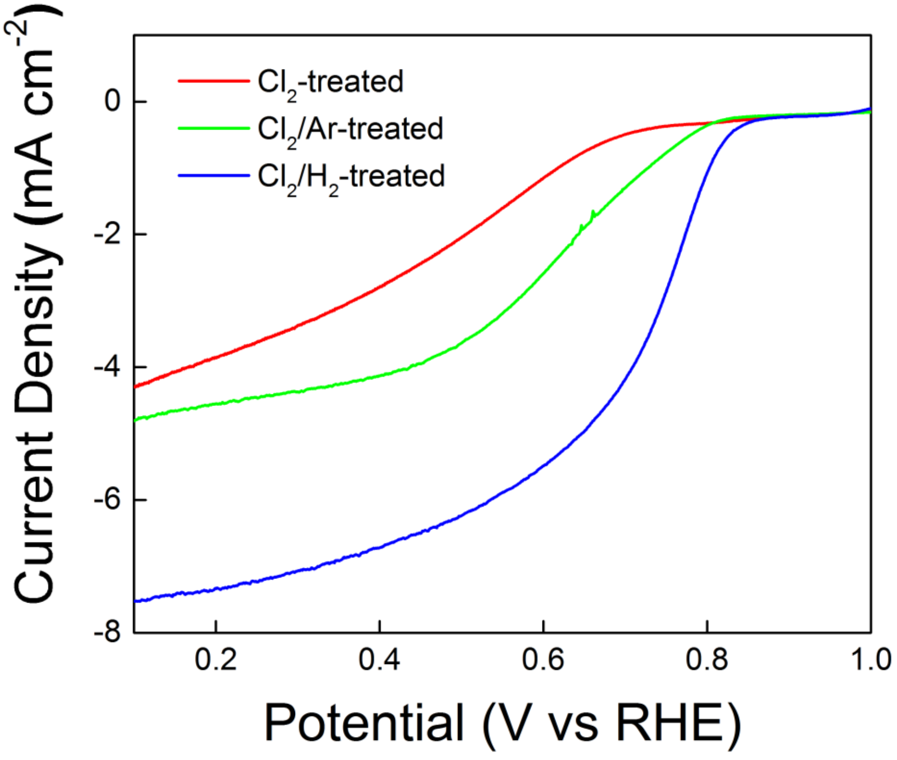

Supplementary Figure 29 | Electrochemical characterization. Cyclic voltammograms for control treatment of Cl_2-treated catalyst with Ar at 900 °C. A slight increase in activity was observed, however complete recovery of catalyst activity is only observed when using H_2. This demonstrates the need for a highly reducing treatment to restore activity.

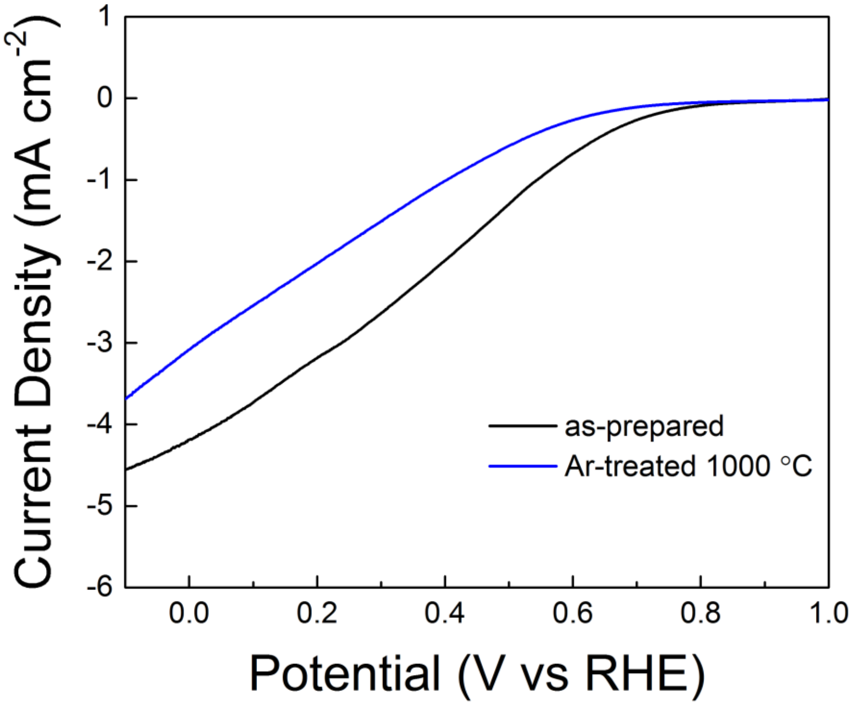

Supplementary Figure 30 | Electrochemical characterization. Cyclic voltammograms for the control treatment of as-prepared catalyst in Ar at 1000 °C. Deactivation of the catalyst was observed which is attributed to the destruction of the active species due to the temperature above that used during catalyst synthesis. In order to prevent temperature effects leading to deactivation, gas treatments were carried out at 900 °C and below.

Supplementary Table 1 | Catalyst selectivity for the reduction of oxygen.

Sample	H_2O_2% (0.3 V vs RHE)
as-prepared	1.59
Cl_2-treated	4.95
H_2-treated	1.83

Supplementary Table 2 | Elemental analysis of catalyst materials. Fe content determined using ICP-OES, Cl content using ion selective electrode, and N content from CHN analysis. Note that the wt% of Fe in the H_2-treated sample is greater than in the other samples in part due to a decrease in total mass cause by the removal of Cl and etching of C during the treatment.

**Sample**	**Fe (wt%)**	**Cl (wt%)**	**N (wt%)**
as-prepared	1.61	0.15	3.39
Cl_2-treated	0.72	8.12	2.15
H_2-treated	2.07	0.06	2.49

Supplementary Table 3 | Mössbauer fitting parameters. Values of Mössbauer fitting obtained for sample spectra for isomer shift (δ_{iso}), quadrupole splitting (ΔE_Q), internal magnetic field (ΔH_{int}) and full-width at half-max (fwhm). Assignments were made by comparing to previous work.

Component	Color	δ_{iso}/ mm s^{-1}	ΔE_Q/ mm s^{-1}	ΔH_{int}/ T	fwhm/ mm s^{-1}	Assignment	Ref.
Doublet 1		0.34	0.95	-	0.88250	FeN_4 / FeN_{2+2}	[2,4-10]
Doublet 2		0.12	0.32	-	0.36525	Fe_xN x < 2	[8,9,11,12]
Sextet 1		0.18	-	19.2	0.47439	Fe_3C	[2,4,10,13]
Sextet 2		0.00	-	32.9	0.45791	α-Fe	[7,10]
Singlet 1		-0.10	-	-	0.41762	spm α-Fe	[2,5-8,13]

Supplementary Table 4 | Mössbauer fitting parameters. Values of Mössbauer fitting for isomer shift (δ_{iso}) and internal magnetic field (ΔH_{int}) for Fe_3S_4 observed in sample treated with H_2 at 600 °C.

Component	Color	δ_{iso}/ mm s^{-1}	ΔH_{int}/ T	Assignment	Ref.
Sextet		0.8	31.0	Fe_3S_4	[2]

Supplementary Table 5 | N 1s peak location assignments. N 1s XPS fitted peak locations and assignment of N species. Range reflects peak locations for all samples shown in this work.

Peak Location	Species Assignment
403.7 – 404.3	Oxydic
401.0 – 401.4	Graphitic
399.3 – 399.6	Pyrrolic
398.1 – 398.3	Pyridinic

Supplementary Table 6 | CHN elemental analysis of N-free catalyst material.

Sample	C (wt%)	H (wt%)	N (wt%)
N-free	0.05	0.00	0.03

Supplementary Table 7 | Elemental analysis results. A comparison of Cl removal using treatment with either H_2 or Ar at 900 °C for 30 min.

Sample	Cl content (wt%)
as-prepared	0.96
Cl_2-treated	9.34
Cl_2/Ar-treated	1.49
Cl_2/H_2-treated	0.75

Supplementary Note 1. Methods for EXAFS data analysis

FEFFIT program[14] was used to analyze EXAFS data in hydrated iron (III) chloride ($FeCl_3 \cdot xH_2O$) and Cl_2-treated catalyst. We obtained theoretical photoelectron scattering amplitudes and phases from ab-initio calculations with FEFF8.5 code[15] for $FeCl_3 \cdot 6H_2O$ model (see inset in **Fig. 4a**).[16] For FEFF calculations the complex exchange-correlation Hedin-Lundqvist potential and default values of muffin-tin radii as provided within the FEFF8.5 code were employed.

Fitting was carried out in *R*-space. Fourier transforms (FTs) were carried out in the range from k_{min} = 2.0 Å^{-1} to k_{max} = 11.0 Å^{-1}. The *R*-range used in the analysis was from 1.0 Å to 2.4 Å.

The composition of the first coordination shell in $FeCl_3 \cdot xH_2O$ and Cl_2-treated catalyst was modeled with two contributions from (i) Cl atoms and (ii) lighter atoms (C, N or O). Because EXAFS analysis cannot discriminate between these three latter backscatters, we represented them all in our calculations by Fe-O pair. Analysis of $FeCl_3 \cdot xH_2O$ was performed first. We assumed that Fe-Cl coordination number in this case is the same as in $FeCl_3 \cdot 6H_2O$, and is equal to 2. Fe-O coordination number, corrections to the model Fe-O and Fe-Cl distances, and the corresponding MSRD factors were allowed to vary in the fits. Additional fitting variable was the correction ΔE to the photoelectron energy origin E_0. The best fit value of the ΔE was obtained to be close to 0. Amplitude reduction factor S_0^2 was found to be approximately equal to 0.85. Both the ΔE and S_0^2 values were subsequently fixed at these values for analysis of the Cl_2-treated catalyst. The fitting parameters were the corrections to the model Fe-O and Fe-Cl distances, their corresponding MSRD values, as well as the corresponding Fe-O and Fe-Cl coordination numbers. The reduced χ^2 values were ca. 78 for $FeCl_3 \cdot xH_2O$ and ca. 94 for Cl_2-treated NPM catalyst material. The obtained values for Fe-Cl interatomic distances for $FeCl_3 \cdot xH_2O$ and Cl_2-treated NPM catalyst material were obtained to be similar, but the coordination numbers were found to be different (**Table 1**).

For the H_2-treated catalyst multiple-scattering analysis was performed using FEFFIT and FEFF8.5 codes. FTs were carried out in the range from k_{min} = 3.0 Å^{-1} up to k_{max} = 12.5 Å^{-1}. The R-range used in the analysis was from 0.8 Å to 5.2 Å. The bcc structure model was used to generate theoretical amplitudes and phases for all paths included in the fit. We included the nearest single-scattering (SS) and the most important multiple-scattering (MS) paths: linear double and triple scattering paths and double-scattering (DS) paths within the first coordination shell. To reduce the number of independent fitting parameters, all half path lengths R_i of all the (SS and MS) paths used in the fit were constrained to be $R_i = r_i\,(1+\varepsilon)$, where r_i is the corresponding value for a model bcc structure and ε is an isotropic lattice expansion/contraction parameter. MSRD factors and coordination numbers for SS contributions were fitted independently. Coordination numbers, distances and disorder parameters in collinear multiple-scattering paths were constrained to be related to those in the corresponding single scattering paths.[17]

For the analysis of EXAFS data for the foil, additional two fitting parameters were included: S_0^2 and ΔE_0. Their best values of 0.67 and 0.7 eV, respectively, obtained for Fe foil data, were then fixed in fits of the data for H_2-treated NPM catalyst material. The reduced χ^2 values were ca. 635 for Fe foil and ca. 609 for the H_2-treated NPM catalyst material.

Supplementary Fig. 15 displays calculated behaviors of the first two coordination numbers of Fe-Fe pairs as a function of nanoparticle size, corresponding to cubic shape and bcc structure of the model particles. The corresponding best fit values for the H_2-treated NPM catalyst, together with their error bars, are shown as well. The intercepts of the experimental values and model curves define the region of sizes where experiment and model agree. For the two coordination shells, these regions overlap in the 1.0–1.5 nm range, which indicates that the Fe particles are, on the average, of 1.0–1.5 nm in size.[17]

Supplementary Note 2. VSM Fitting

VSM data was fit using the Langevin function for superparamagnetic particles given by:

$$\frac{M}{M_{sat}} = L\left(\frac{M_s V H}{k_B T}\right) \tag{1}$$

where M is equal to the magnetization (emu g^{-1}), M_{sat} is equal to the saturation magntization (emu g^{-1}), M_s is equal to the spontaneous magnetization determined for Fe using the magnetic moment of an Fe atom in metallic Fe (2.2 μ_B) and the volume of a BCC Fe unit cell (emu cm^{-3}), V is equal to the volume of a particle (cm^3), H is equal to the applied magnetic field, k_B is the Boltzmann constant (cm^2 g s^{-2} K^{-1}), and T is the temperature (K). $L(x) = \coth(x) - 1/x$ is the Langevin function. Average particle diameter was calculated by determining the value of V for each sample and assuming spherical Fe particles.

Supplementary Note 3. Characterization of catalysts before and after ORR operation

In order to investigate the possibility of the formation of new active species during ORR the as-prepared and H_2-treated catalysts were investigated during and after operation. The electrochemical activity was unchanged during operation as observed by CV (**Supplementary Fig. 25)**. XPS obtained after ORR operation exhibits a decrease in pyridinic N and an increase in pyrrolic N while the oxydic and graphitic N remain (**Supplementary Fig. 26**). This result again suggests that the pyridinic N species are not required for ORR. Vibrating sample magnetometry (VSM) performed before and after ORR operation shows a decrease in the magnetization due to the dissolution of unprotected surface species while the signature of small superparamagnetic particles is maintained (**Supplementary Fig. 27**). In order to mitigate any effects from putative dissolved Fe, both catalysts were run in electrolyte solution containing up to 100 mM of $ZnClO_4$ and $MnClO_4$. The excess of Zn and Mn, which are ORR inactive metals, should fill the vacant N sites and prevent any dissolved Fe from coordinating. Using CV, no effect on the ORR activity was observed with either Zn or Mn in the electrolyte (**Supplementary Fig. 28**). Together, the electrochemical tests along with XPS and VSM show that there were no new Fe or Fe-N species formed during operation and suggest that the Fe particles encapsulated by C and graphitic N are responsible for the observed activity and stability of NPM catalysts.

Supplementary References

1 Faubert, G. *et al.* Activation and characterization of Fe-based catalysts for the reduction of oxygen in polymer electrolyte fuel cells. *Electrochim. Acta* **43,** 1969-1984, (1998).

2 Ferrandon, M. *et al.* Multitechnique characterization of a polyaniline–iron–carbon oxygen reduction catalyst. *J. Phys. Chem. C* **116,** 16001-16013, (2012).

3 Battino, R., Rettich, T. R. & Tominaga, T. The solubility of oxygen and ozone in liquids. *J. Phys. Chem. Ref. Data* **12,** 163-178, (1983).

4 Blomquist, J., Lang, H., Larsson, R. & Widelov, A. Pyrolysis behaviour of metalloporphyrins. Part 2.-a mossbauer study of pyrolysed FeIII tetraphenylporphyrin chloride. *J. Chem. Soc. Faraday Trans.* **88,** 2007-2011, (1992).

5 Koslowski, U. I., Abs-Wurmbach, I., Fiechter, S. & Bogdanoff, P. Nature of the catalytic centers of porphyrin-based electrocatalysts for the ORR: A correlation of kinetic current density with the site density of Fe−N4 centers. *J. Phys. Chem. C* **112,** 15356-15366, (2008).

6 Kramm, U. I. *et al.* New insight into the nature of catalytic activity of pyrolysed iron porphyrin based electro-catalysts for the oxygen reduction reaction (ORR) in acidic media. *ECS Trans.* **25,** 93-104, (2009).

7 Kramm, U. I. *et al.* Influence of the electron-density of FeN4-centers towards the catalytic activity of pyrolyzed FeTMPPCl-based ORR-electrocatalysts. *J. Electrochem. Soc.* **158,** B69-B78, (2011).

8 Kramm, U. I., Herrmann-Geppert, I., Bogdanoff, P. & Fiechter, S. Effect of an ammonia treatment on structure, composition, and oxygen reduction reaction activity of Fe–N–C catalysts. *J. Phys. Chem. C* **115,** 23417-23427, (2011).

9 Kramm, U. I. *et al.* Structure of the catalytic sites in Fe/N/C-catalysts for O2-reduction in PEM fuel cells. *Phys. Chem. Chem. Phys.* **14,** 11673-11688, (2012).

10 Zitolo, A. *et al.* Identification of catalytic sites for oxygen reduction in iron- and nitrogen-doped graphene materials. *Nat. Mater.* **14,** 937-942, (2015).

11 Borsa, D. M. & Boerma, D. O. Phase identification of iron nitrides and iron oxy-nitrides with mössbauer spectroscopy. *Hyperfine Interact.* **151-152,** 31-48, (2003).

12 Schaaf, P. Laser nitriding of metals. *Prog. Mater. Sci.* **47,** 1-161, (2002).

13 Schulenburg, H. *et al.* Catalysts for the oxygen reduction from heat-treated iron(III) tetramethoxyphenylporphyrin chloride: Structure and stability of active sites. *J. Phys. Chem. B* **107,** 9034-9041, (2003).

14 Newville, M. *et al.* Analysis of multiple-scattering XAFS data using theoretical standards. *Physica B Cond. Matter* **208–209,** 154-156, (1995).

15 Ankudinov, A. L., Ravel, B., Rehr, J. J. & Conradson, S. D. Real-space multiple-scattering calculation and interpretation of x-ray-absorption near-edge structure. *Phys. Rev. B* **58,** 7565-7576, (1998).

16 Lind, M. D. Crystal structure of ferric chloride hexahydrate. *The Journal of Chemical Physics* **47,** 990-993, (1967).

17 Frenkel, A. Solving the structure of nanoparticles by multiple-scattering EXAFS analysis. *J. Synchrotron Rad.* **6,** 293-295, (1999).
